# Biochemical characterization of a multiple prenyltransferase from *Tolypocladium inflatum*

**DOI:** 10.1007/s00253-024-13113-6

**Published:** 2024-03-26

**Authors:** Haiyan Han, Shuang Peng, Qian Wang, Hongwei Wang, Pengchao Wang, Chang Li, Jianzhao Qi, Chengwei Liu

**Affiliations:** 1 Key Laboratory for Enzyme and Enzyme-Like Material Engineering of Heilongjiang, College of Life Science, Northeast Forestry University, No. 26 Hexing Road, Harbin, 150040 Heilongjiang China; 2 Department of Medicinal Chemistry and Natural Medicine Chemistry, College of Pharmacy, Harbin Medical University, Harbin, 150040 China; 3 Shaanxi Key Laboratory of Natural Products & Chemical Biology, College of Chemistry & Pharmacy, Northwest A&F University, No.3 Taicheng Road, Yangling, 712100 China

**Keywords:** Prenyltransferase, Indole diterpene, *Tolypocladium inflatum*, Paxilline, Structural diversity

## Abstract

**Abstract:**

Prenylation plays a pivotal role in the diversification and biological activities of natural products. This study presents the functional characterization of TolF, a multiple prenyltransferase from *Tolypocladium inflatum*. The heterologous expression of *tolF* in *Aspergillus oryzae*, coupled with feeding the transformed strain with paxilline, resulted in the production of 20- and 22-prenylpaxilline. Additionally, TolF demonstrated the ability to prenylated the reduced form of paxilline, β-paxitriol. A related prenyltransferase TerF from *Chaunopycnis alba*, exhibited similar substrate tolerance and regioselectivity. In vitro enzyme assays using purified recombinant enzymes TolF and TerF confirmed their capacity to catalyze prenylation of paxilline, β-paxitriol, and terpendole I. Based on previous reports, terpendole I should be considered a native substrate. This work not only enhances our understanding of the molecular basis and product diversity of prenylation reactions in indole diterpene biosynthesis, but also provides insights into the potential of fungal indole diterpene prenyltransferase to alter their position specificities for prenylation. This could be applicable for the synthesis of industrially useful compounds, including bioactive compounds, thereby opening up new avenues for the development of novel biosynthetic strategies and pharmaceuticals.

**Key points:**

• *The study characterizes TolF as a multiple prenyltransferase from Tolypocladium inflatum.*

• *TerF from Chaunopycnis alba shows similar substrate tolerance and regioselectivity compared to TolF.*

• *The research offers insights into the potential applications of fungal indole diterpene prenyltransferases.*

**Graphical abstract:**

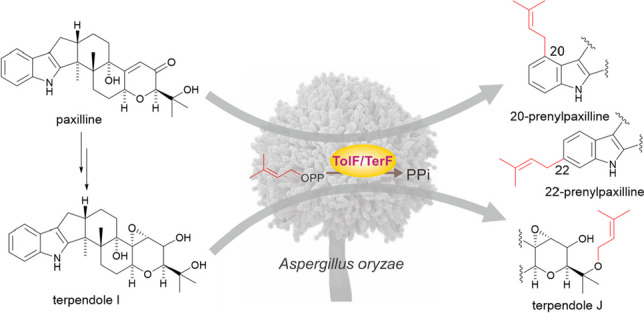

**Supplementary Information:**

The online version contains supplementary material available at 10.1007/s00253-024-13113-6.

## Introduction

Indole diterpenes (IDTs) constitute a class of structurally unique natural products derived from fungi. Their distinguishing characteristic involves the attachment of a C20 long-chain geranyl–geranyl diphosphate (GGPP) to the indole ring, followed by subsequent modifications, including cyclization, oxidation, prenylation, and other modifications, resulting in structurally complex compounds (Ozaki et al. 2023). These compounds are primarily derived from fungal species such as *Penicillium* sp., *Aspergillus* sp., and *Claviceps* sp., among others (Reddy et al. 2019). Based on their structural features, IDTs can be broadly classified into two major types: paspaline and non-paspaline, because paspaline is characterized by the presence of six rings, therefor, starting from the indole ring side, these six rings are sequentially named from A to F (Niu et al. 2023; Saikia et al. 2008). To date, over 200 IDT compounds have been reported, with paspaline-type IDTs accounting for more than 70% of the total (Niu et al. 2023).

**Fig. 1:**
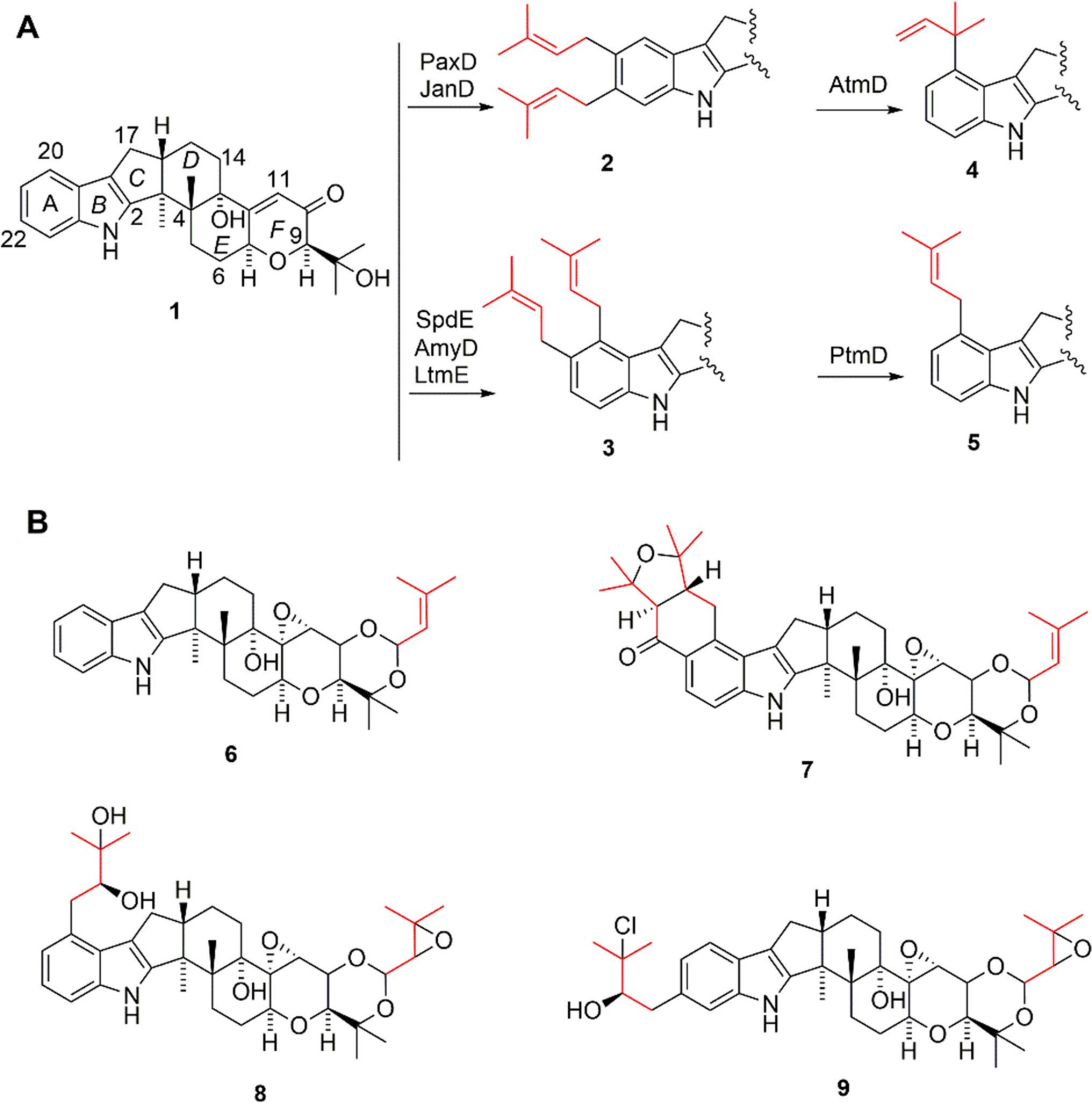
Typical prenylation IDTs. **A** the product resulting from PT catalysis with **1** as a substrate; **B** the prenylation of IDTs' A and/or F rings

Prenylation is one of the major factors contributing to the diversity of IDT compounds, including modifications on the A-ring and F-ring. The diversity is further reflected in the different forms of attachments, such as regular and reverse, the positions and number of attachments. These modifications greatly enhance the structural diversity of IDTs (Hibbard et al. 2023; Oikawa et al. 2016; Richardson et al. 2022; Van de Bittner et al. 2018). The first characterization of the extracellular enzymatic activity of IDT prenyltransferase (PT) was performed on PaxD, which was shown to catalyze the regular addition of prenyl groups at positions 21 and 22 of paxilline (**1**) to give 21,22-diprenylpaxilline (**2**) (Liu et al. 2014a). Subsequent studies showed that JanD had the same activity (Liu et al. 2016), however, LtmE, SpdE and AmyD reacted at positions 20 and 21 of the **1** to produce 20, 21-diprenylpaxilline (**3**) (Jiang et al. 2020; Kudo et al. 2018; Liu et al. 2014b). For the same substrates, the product of the AtmD was reversely-20-prenylpaxilline (**4**) (Liu et al. 2013), and the activity of PtmD catalyzes the production of 20-prenylpaxilline (**5**) is very weak (Fig. 1A) (Liu et al. 2015). In addition to A-ring modifications, the F-ring also undergoes prenylation, such as in terpendole C (**6**), lolitrem B (**7**), tolypocladin I (**8**), and tolypocladin J (**9**) (Fig. 1B). Recently, a series of compounds named tolypocladin A-L (Fig. S1) was identified in fungus *Tolypocladium* sp., featuring prenylation modifications in the A and F rings (Xu et al. 2019a, 2019b). However, the genes and enzymes involved in the biosynthesis of tolypocladins and their precise functions remain unknown.

Through genomic informatics analysis, we discovered a gene cluster in *Tolypocladium* sp. sharing approximately 70% similarity with the *ter* gene cluster, a known terpendole biosynthetic gene cluster (Motoyama et al. 2012). We conducted functional analysis of PT for TolF within this cluster. When *tolF* was expressed in *A. oryzae* and feeding with substrate **1**, two new compounds were generated. Through nuclear magnetic resonance (NMR) and mass spectrometry analysis, these compounds were identified as 20- and 22-prenylpaxilline (**5**, **10**). Consistent results were obtained through in vitro enzyme assays. When terpendole I (**11**) was used as the substrate, both in vivo feeding and in vitro enzyme reactions yielded a single product, with a molecular weight matching that of terpendole J (**12**). We confirmed that TerF also produces the same compounds when reacting with **1**. These results indicate that the native substrate for both enzymes is likely to be **11**. We also synthesized the reduced product of **1**, β-paxitriol (**13**), and both in vivo and in vitro experiments demonstrated that TolF and TerF can introduce prenyl group at either the 20 or 22 positions of β-paxitriol (**14**, **15**); they exhibit broad substrate tolerance. These outcomes illustrate the broad substrate selectivity of PT, contributing to a deeper understanding of the versatility of IDTs.

## Material and methods

### General methods

All reagents used in this study were purchased from the commercially available. Oligonucleotides required for polymerase chain reaction (PCR) were procured from Tsingke Biotechnology Co., Ltd.

The Diode array detector (DAD) on Agilent 1260 Infinity II (Agilent Technologies, USA) was used for High-performance liquid chromatography (HPLC) analysis. Liquid Chromatography-Mass Spectrometry (LC–MS) data were acquired with an AB SCIEX Triple TOF 6600 instrument. Proton and carbon nuclear magnetic resonance (NMR) spectra were obtained using a Bruker AVANCE III HD 500 spectrometer. The chemical shifts were reported in ppm on the δ scale, using CDCl_3_ as an internal reference (^1^H NMR = 7.26 ppm, ^13^C NMR = 77.16 ppm).

### Microbial strains and culture conditions

The strain *T. inflatum* CICC-2598, sourced from the China Center of Industrial Culture Collection, which was propagated in a liquid medium of potato dextrose broth (PDB) at a temperature setting of 25℃ and an agitation speed of 170 rpm over a week. Then, the genome was extracted for the cloning of the *tolF* gene. The *A. oryzae* NSAR1 (Jin et al. 2004) strain was utilized as the heterologous expression host, and was grown in a DPY medium (containing 2% dextrin, 1% polypeptone, and 0.5% yeast extract in a 100 mL solution) enriched with suitable nutrients, at a temperature of 30℃ and a rotation speed of 200 rpm (Tagami et al. 2013). Conventional DNA engineering was executed with *E. coli* DH5α, while protein expression was facilitated using *E. coli* BL21. All *E. coli* strains were cultured at a temperature of 37℃, whereas protein expression was performed at 16℃.

### Isolation of genomic DNA and assembly of plasmid

The genomic DNA of *T. inflatum* were performed as described previously (Tagami et al. 2013). The *tolF* gene (GenBank: OR966868) was amplified from the *T. inflatum* genome using gene-specific primers (Table S3). The amplified gene was then incorporated into the pUARA2 vector, resulting in the creation of the pUARA2-*tolF* expression plasmid. Similarly, the *terF* gene (GenBank: AB725916) was synthesized by Tsingke Biotechnology Co., Ltd., and was also inserted into the pUARA2 vector using the same method, leading to the formation of the pUARA2-*terF* plasmid.

### Transformation of* A. oryzae*

The *A. oryzae* transformation was conducted using the protoplast-polyethylene glycol technique, as outlined in the work of Tagami et al. (Tagami et al. 2013). The transformation process employed the plasmids pUARA2-*tolF* and pUARA2-*terF*, leading to the creation of AO-*tolF* and AO-*terF*, respectively.

### Biotransformation of *A. oryzae *transformants

The transformant AO*-tolF* or AO*-terF* were introduced into a nutrient-rich MPY medium (comprising 3% malotose, 0.5% yeast extract and 1% polypeptone) and added the appropriate nutrients (2 mL) in a 10 mL test tube. A methanol solution of the substrate (20 μg) was subsequently added to the culture medium. The culture was then incubated for an additional three days at 30℃, under a rotation speed of 200 rpm. Following incubation, the fermentation broth was immersed in acetone and left overnight at ambient temperature. The mixture was then filtered, and the filtrate was concentrated under vacuum. The residual water by extracted with ethyl acetate, and the organic layers were also concentrated under vacuum. Finally, the samples were analyzed using HPLC and LC–MS.

### Cloning, overexpression, and purification of TolF and TerF

Total RNA was extracted from AO*-tolF* mycelium sample using TRIzol reagent (Invitrogen) according to the manufacturer’s instructions. The first strand cDNA was then synthesized using the PrimeScript™ II First Strand cDNA Synthesis Kit (TAKARA) according to the manufacturer's instructions. Intron less DNA clones of *tolF* were amplified from the cDNA of AO*-tolF* and inserted into BamHI-digested pMAL-c5x to construct the pMal-c5x-*tolF* expression plasmid. The pMal-c5x-*tolF* plasmid was then transformed into *E. coli* BL21 for overexpression. The transformants were incubated in LB medium (200 mL) supplemented with 100 μg/mL ampicillin grown at 37℃ and 200 rpm to an optical density of 0.6 at 600 nm, then cooled to 16℃. Protein expression was induced by adding isopropyl-β-D-thiogalactopyranoside (IPTG) to a final concentration of 0.2 mM, followed by further incubation for about 20 h at 16℃ and 200 rpm. The cells containing were harvested and treated as follows. The cells of TolF were resuspended in 4 mL buffer A (50 mM Tris–HCl, 500 mM NaCl, 10% glycerol, pH 7.5) and were lysed by sonication on ice. Cellular debris was removed by centrifugation at 15,000 rpm, at 4℃, for 10 min and purified using MBPTrap HP columns. The purified enzymes were checked by SDS-PAGE. Finally, the purified protein was quickly frozen in liquid nitrogen and saved at -80℃. The cloning, overexpression, purification, and storage methods for TerF are the same as those described above.

### In vitro assay of TolF and TerF

The enzymatic reaction of TolF or TerF with **1**, **11,** and **13** was performed in 100 μL of reaction mixtures containing 50 mM Tris–HCl buffer (pH 7.0), 1 mM of substrates, 1 mM DMAPP, 5 mM MgCl_2_, and 5 μg enzyme at 30℃ overnight. The reaction was terminated by adding 100 μL of methanol and vortex mixing. The supernatant obtained after centrifugation was analyzed by HPLC and LC–MS.

## Analysis of the metabolites

The metabolites were examined using an HPLC system equipped with an Agilent TC-C18 column (250 mm × 4.6 mm). The conditions were as follows: from 0 to 5 min, 60% of solvent B was used; from 5 to 25 min, a linear gradient from 60 to 100% of solvent B was applied; and from 25 to 30 min, 100% of solvent B was used. The mobile phase consisted of solvent A (H_2_O) and solvent B (CH_3_OH), with a flow rate of 1 mL/min. The samples were further analyzed using a TripleTOF 6600 mass spectrometer (AB/SCIEX, Milford, MA) in conjunction with an HPLC system (AB/SCIEX). Chromatographic separation was achieved using a Kinetex C18 100A column (Phenomenex) with dimensions of 150 mm × 4.6 mm and a particle size of 2.6 μm. The conditions were as follows: from 0 to 10 min, a gradient from 5 to 100% of solvent B was applied; and from 10 to 15 min, 100% of solvent B was used. The mobile phase consisted of solvent A (H_2_O + 0.1% formic acid) and solvent B (CH_3_CN + 0.1% formic acid), with a flow rate of 0.6 mL/min.

### Isolation and purification of metabolite

The transformant AO-*tolF* was inoculated into a MPY medium, supplemented with the necessary nutrients, and a total of 2 L. Subsequently, 2 mg of Substrate **1** or **13**, dissolved in methanol, was added to the 100 mL MPY medium. The mixture was then incubated for an additional 3 days at 30℃, under a rotation speed of 200 rpm. The mycelium was obtained by filtration and extracted by acetone. The mycelium was obtained by filtration and extracted by acetone to obtain the bold substance. The crude extract was re-extracted by ethyl acetate. The ethyl acetate layer was separated using silica gel column chromatography, with a hexane to ethyl acetate ratio ranging from 6:1 to 2:1. The compounds isolated from this process were further purified using semi-preparative HPLC.

## Results

### Identifying the *tol* gene cluster in *T. inflatum*

In 2018, genomic data of *T. inflatum* was publicly disclosed (Bushley et al. 2013). In this study, we acquired a strain from the China Center of Industrial Culture Collection (No. CICC-2598). Initially, we conducted tests to confirm its ability to produce IDT compounds. The fungal strain was cultivated on a rice-based medium, followed by extraction using ethyl acetate. Subsequently, LC–MS analysis was performed to detect and characterize the compound. The results of the molecular network analysis revealed the detection of compounds with molecular weights of 504, 522, and 588 (Fig. S2), characteristic of IDTs. This observation provides evidence for the IDT-producing potential of the CICC-2598 strain, indicating that this strain contains a gene cluster for the synthesis of IDT.

**Fig. 2:**
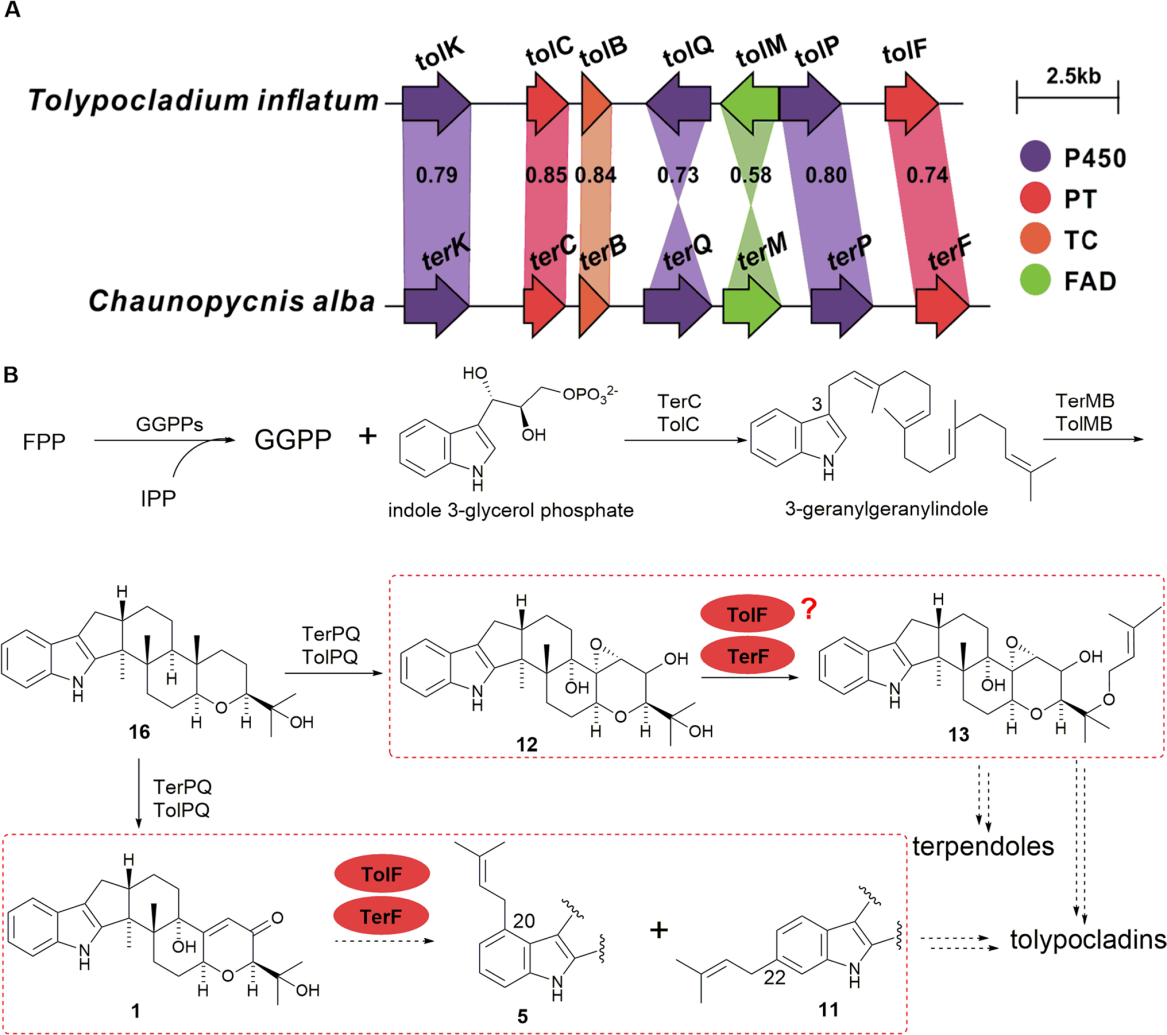
Comparison of *tol* and *ter* gene cluster (**A**), and proposed biosynthetic pathways of terpendoles and tolypocladins (**B**). P450: Cytochrome P450; PT: prenyltransferase; TC: terpene cyclases; FAD: flavin dependent enzyme

Through genome informatics analysis, we identified a *tol* gene cluster in *T. inflatum*, which display similarities with the *ter* gene cluster, a known terpendole biosynthetic gene cluster (Fig. 2A). The *tol* gene cluster contains seven genes, of which *tolCMB* has at least 58% identity with its homologous gene sequence (*terCMB*) (Fig. 2B), which is responsible for the core synthesis of paspaline (**16**) (Motoyama et al. 2012). Two P450 genes, named *tolPQ*, exhibit 80% and 73% identity with *terPQ*, respectively. The PT of TolF shares 74% identity with TerF, indicating a high level of similarity between these two related enzymes.

The structures of tolypocladin IDTs differ from those of terpendoles. Notably, the former has a single prenyl group on the A-ring in addition to prenylation at the OH (F-ring) side. While TerF from the *ter* gene cluster is responsible for prenylation of the F-ring alone (Jiang et al. 2020; Motoyama et al. 2012). However, there is only one PT transferase in the *tol* gene cluster. Therefore, it remains to be determined whether TolF is capable of prenylation of both the A- and F-ring and adding PT modification simultaneously, which requires further investigation.

### Functional analysis of TolF

**Fig. 3:**
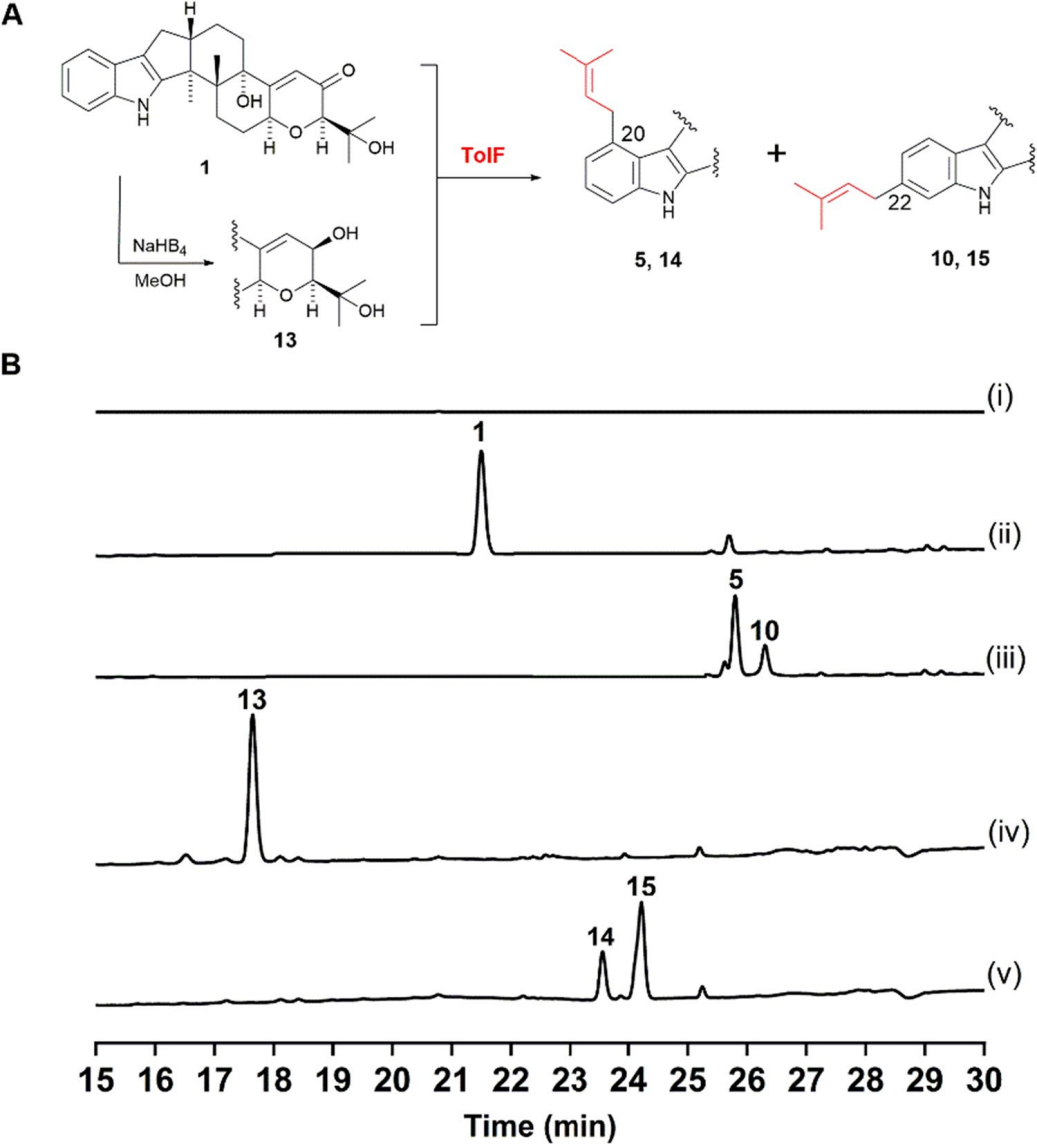
AO*-tolF* feeding with** 1** and **13**. **A** Schematic of the prenylation by TolF. **B** HPLC analytical data: (i) AO-WT; (ii) AO-WT + **1**; (iii) AO*-tolF* + **1**; (iv) AO*-WT* + **13**; (v) AO*-tolF* + **13**

To analyze the functions of PT for TolF within *tol* cluster, the gene was amplified from *T. inflatum* genomic DNA, the purified PCR fragment was cloned into pUARA2 under the control of promoter amyB, then transferred to the heterologous expression host *A. oryzae* NSAR1 to form AO*-tolF* transformants (Tagami et al. 2013). The ethyl acetate extract of the transformed mycelium was analyzed by HPLC. Two new peaks were observed in the AO*-tolF* strain when fermented in MPY medium with feeding **1**. By contrast, these peaks were absent in the wild-type (WT) strain (Fig. 3, trace iii).

Through high-resolution electrospray ionization mass spectrometry (HR-ESI–MS) analysis, the molecular mass of these two new peaks were determined to be C_32_H_41_NO_4_ (m/z 504.3105 and 504.3106 [M + H]^+^), respectively (Fig. S3). Because the molecular weights of both two compounds are 68 Da greater than that of **1** with an [M + H]^+^ ion at m/z 436, this data strongly suggested that these two products were mono-prenylated **1**. To determine the structures of these two new compounds, the mycelial ethyl acetate extract of AO*-tolF* with **1** was obtained from a scaled-up fermentation. The crude extract was purified with silica gel column chromatography and partitioned with hexane–ethyl acetate.

Compound **5** of ^1^H-NMR spectra exhibited new signals for regular prenyl moieties at *δ* = 5.41 (*t*, *J* = 7.9 Hz, 1H), and *δ* = 3.61 (*d*, *J* = 7.0 Hz, 2H), Compound **10** of ^1^H-NMR spectra exhibited at *δ* = 5.36 (*t*, *J* = 7.2 Hz, 1H), and *δ* = 3.41 (*d*, *J* = 7.2 Hz, 2H). Finally, thorough NMR data analysis, encompassing ^1^H-NMR, ^13^C-NMR, COSY (Correlation Spectroscopy), HSQC (Heteronuclear Single Quantum Coherence), HMBC (Heteronuclear Multiple Quantum Coherence), and NOESY (Nuclear Overhauser Effect Spectroscopy), confirmed the structure as 20- and 22-prenylated **1** (Table S1, Fig. S9-S20).

PT transferases often display a broad range of substrate promiscuity, as exemplified by enzymes such as PaxD and AtmD, exhibiting enzymatic activity towards intermediates of various biosynthetic pathways (Liu et al. 2013). Accordingly, we conducted a study on the substrate promiscuity of TolF. Specifically, we synthesized the reduced product of **1**, known β-paxitriol as **13** (Fig. S21) (Miles et al. 1992). When feeding this compound into the AO*-tolF* strain, two distinct products were observed through HPLC analysis (Fig. 3, trace v). HR-ESI–MS analysis determined the molecular weight [M + H]^+^ 506.3264 and 506.3262 of those two compounds, with the presumed molecular formula of C_32_H_43_NO_4_ (calculated as [M + H]^+^ 506.32655). Moreover, a specific fragment of 198.13 was detected by LC–MS analysis (Fig. S4). This fragment is the characteristic peak of the A-ring mono-prenylated form of IDT (Uhlig et al. 2009). Next, we scaled up fed **13** to the AO-*tolF* strain and performed purification of these two compounds using HPLC. One of the compounds exhibited ^1^H data (Fig. S22) that matched with the previously reported 20-prenylpaxitriol (**14**) (Liu et al. 2015). Based on the ^1^H NMR data (Fig. S23), and identification results mentioned earlier, we conclude that the structure of the other compound was 22-prenylpaxitriol (**15**).

### Functional analysis of TerF

**Fig. 4:**
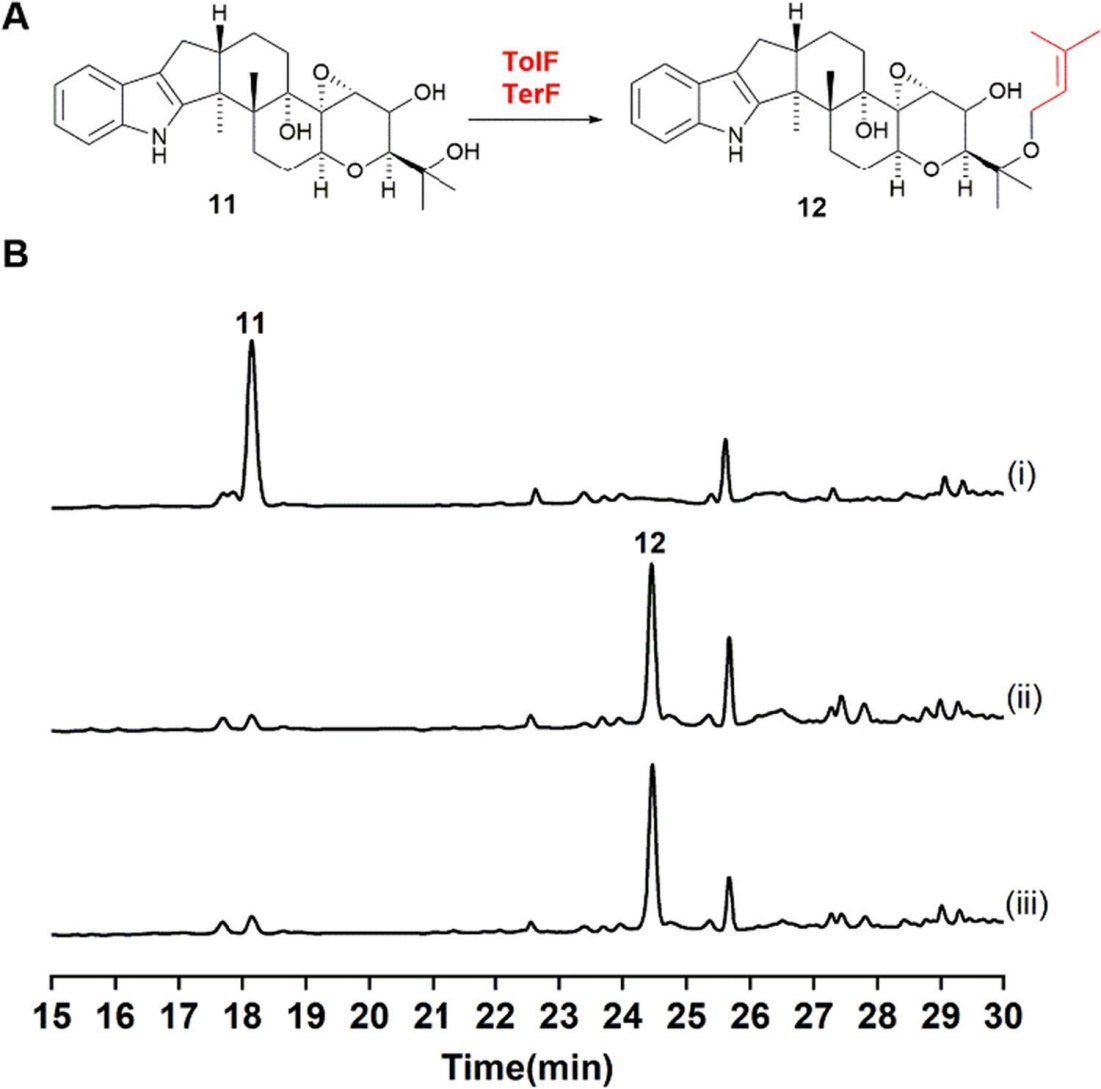
AO*-tolF* and AO*-terF* feeding with **11**. **A** Schematic of the prenylation by TolF and TerF. **B** HPLC analytical data: (i) AO-WT + **11**; (ii) AO*-terF* + **11**; (iii) AO*-tolF* + **11**

As observed in the previous studies, the function of TerF introduced the prenyl group at the OH position of the F-ring in **11** (Jiang et al. 2020; Motoyama et al. 2012). In this study, we constructed a strain of *A. oryzae* expressing *terF*. Because the producing strain was unavailable, we synthesized the gene fragment and constructed the expression plasmid pUARA2-*terF*, which was then introduced into *A. oryzae* to establish the AO*-terF* strain. When this transformed strain was feeding with **11**, a single product (Fig. 4B trace ii) with a molecular weight consistent with the introduction of a prenyl group was detected (Fig. S5). Based on previous reports (Jiang et al. 2020; Motoyama et al. 2012), this compound is **12**. However, when AO*-terF* was co-cultured with **1**, two compounds identical to those produced by TolF were observed, with identical retention times and LC-MS patterns to **5** and **10** (Fig. S6).

Additionally, we conducted experiments feeding **11** to the AO*-tolF* strain, and similarly observed only a single product (Fig. 4B trace iii). These results indicate that **11** is the native substrate of TerF and TolF. Furthermore, both TolF and TerF exhibit similar functions and broad substrate specificity. However, within the *tol* biosynthetic gene cluster, only one PT might be responsible for the reaction with multiple substrates.

### In vitro characterization of TolF and TerF

To elucidate the specific roles of TolF, we aimed to employ recombinant TolF for in vitro assays. The predicted gene product consists of a sequence of 433 amino acids (Table S2). The open reading frame of *tolF* was amplified using cDNA as a template, and subsequently cloned into the pCold I, pQE30, and pET28a expression vector, however, no soluble proteins were obtained. As a potential solution, we endeavored to optimize the codon usage of the gene, to achieve a soluble protein after overexpression. Unfortunately, despite these efforts, obtaining soluble protein was still unattainable.

In a previous study conducted by our research in 2014, we successfully expressed and purified AmyD, a fungal prenyltransferase that catalyzes the regular diprenylation of paxilline sites 20 and 21, achieved using the maltose-labeled expression vector pMAL-c5x (Liu et al. 2014b). Therefore, our focus shifted toward constructing the TolF gene in the pMAL-c5x vector. Remarkably, subsequent overexpression resulted in the successful production of soluble protein. The homogeneity of the protein was ensured through purification using a one-step MBPTrap HP chromatography process. The obtained recombinant TolF, with a calculated molecular mass of 47 kDa, was further validated for its molecular size and purity through SDS-PAGE analysis (Fig. S7). We also expressed the synthesized *terF* gene in *E. coli* and purified the TerF enzyme for in vitro enzymatic reactions.

**Fig. 5:**
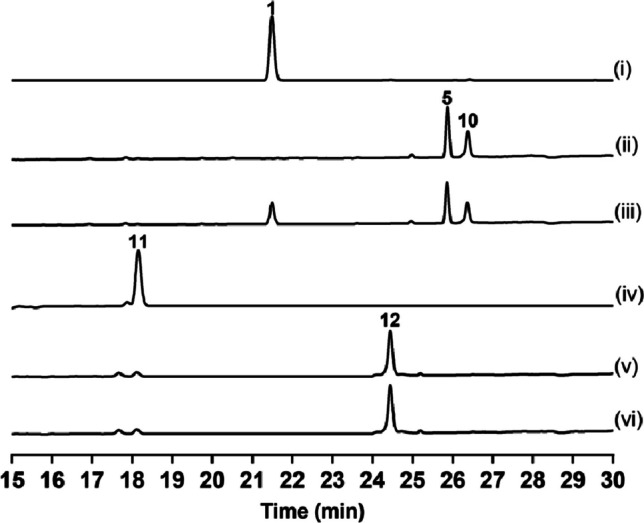
In vitro characterization of TolF and TerF: (i) (iv) without enzyme; (ii) TolF with **1** and DMAPP; (iii) TerF with **1** and DMAPP; (v) TolF with **11** and DMAPP; (vi) TerF with **11** and DMAPP

Subsequently, we analyzed the catalytic activity of the purified recombinant enzyme products using HPLC and LC–MS. As expected, the formation of **5** and **10** was observed when TolF or TerF was incubated with **1** and DMAPP (Fig. 5, traces ii and iii), and two compounds **14** and **15** were detected when **13** was used as a substrate (Fig. S8), while controls without the enzyme did not lead to the formation of these compounds. However, when **11** was used as the prenyl acceptor, only a single product **12** was observed (Fig. 5, trace v and vi).

## Discussion

In this study, we identified a putative indole diterpene biosynthetic gene cluster *tol* consisting of seven genes in the genome of *T. inflatum*. Within this gene cluster, a multiple PT was identified, named TolF, which shares 74% amino acid identity with the known enzyme TerF involved in the F-ring modification of IDTs (Motoyama et al. 2012). Detailed analysis of both enzymes was conducted through heterologous expression in *A. oryzae* and in vitro enzymatic assays. Experimental results revealed that TolF and TerF could catalyze the production of 20- and 22-position prenylated products of **1**, respectively. In order to investigate the broad substrate specificity of these two enzymes, the reduced product of **1**, the compound **13** was synthesized. When **13** as substrate both in vivo and in vitro experimental analyses detected two new products. By LC–MS analysis, both compounds have a specific fragment 198.13, which is produced by C-ring cleavage of mono-prenylated paxilline (Liu et al. 2014a). In combination with the ^1^H NMR data and TolF catalyzed **1** production of **5** and **10**, these two new compounds should be catalyzed in the same position generated to **14** and **15**. However, when compound **11** was used as a substrate, only one product was produced, which is consistent with previous reports. In 2012, Motoyama et al. proposed that TerF function is to modify the hydroxyl group on the F-ring by knocking out the gene in the strain *Chaunopycnis alba* (Motoyama et al. 2012). In 2020, we used heterologous expression in *A. oryzae* and produced terpendole C when the *terFK* genes were introduced into a strain producing compound **11**, this result showed that compound **12** was definitively identified as an intermediate (Jiang et al. 2020). In the present study, our experimental results further support this conclusion. Although both enzymes exhibit broad substrate specificity, we found that under the same substrate concentration and reaction time conditions, those two enzymes by in vitro, when reacting with compound **11** the substrate was completely consumed, while when reacting with compounds **1** and **13**, the substrate there was still some residual substrate, the result indicating that the native substrate in the cell was **11**.

These findings expand the substrate promiscuity of PT transferases and the diversity of IDT compounds, providing insights into the biosynthetic pathway of tolypocladins compounds. The broad substrate tolerance of these enzymes introduces the potential for their application in combinatorial biosynthesis strategies. By integrating these enzymes with various pathways or engineering enzymes with new catalytic activities, it becomes feasible to generate unique compounds with potential beneficial properties. This approach could lead to the discovery of new bioactive molecules, expanding the repertoire of available natural products and potentially contributing to the development of new therapeutic agents.

## Supplementary Information

Below is the link to the electronic supplementary material.

Supplementary file1 (PDF 2198 KB)

## Data Availability

All data generated during this study are included in this published article and its supplementary information file.
