# Supplementary material for: Biochemical characterization of a multiple prenyltransferase from Tolypocladium inflatum
Source: Appl Microbiol Biotechnol. 2024 Mar 26;108(1):275. doi: 10.1007/s00253-024-13113-6 (PMC10965706; doi:10.1007/s00253-024-13113-6)
Supplement: Supplementary file 1 — Supplementary file1 (PDF 2198 KB) [file 253_2024_13113_MOESM1_ESM.pdf]

Supporting Information

Biochemical Characterization of a Multiple Prenyltransferase from *Tolypocladium inflatum*

Haiyan Han[1] · Shuang Peng[1] · Qian Wang[1] · Hongwei Wang[1] · Pengchao Wang[1] · Chang Li[2] · Jianzhao Qi [1,3]* · Chengwei Liu [1]*

[1] Key Laboratory for Enzyme and Enzyme-like Material Engineering of Heilongjiang, College of Life Science, Northeast Forestry University, Harbin 150040, Heilongjiang, China.

[2] Department of Medicinal Chemistry and Natural Medicine Chemistry, College of Pharmacy, Harbin Medical University, Harbin 150081, China

[3] Shaanxi Key Laboratory of Natural Products & Chemical Biology, College of Chemistry & Pharmacy, Northwest A&F University, Yangling 712100, China

Corresponding Authors

Jianzhao Qi

*Tel: +86-29-87092335; E-mail: qjz@nwafu.edu.cn.

Chengwei Liu

*Tel: +86-451-82191378; E-mail: liuchw@nefu.edu.cn.

Contents

Supplementary experimental section 3

Supporting Tables:

Table S1 ^{1}H and ^{13}C NMR data of the compounds 20-prenylpaxilline (**5**) and 22-prenylpaxilline (**10**). 4

Table S2 DNA and protein sequences of tolF. Highlighted in yellow are regions predicted to be introns in this study 5

Table S3 Plasmids used in this study. 6

Supporting Figures

Fig. S1 Chemical structure of tolypocladin A-L. 7

Fig. S2 Molecular network of the metabolic products from *T. inflatum*. 8

Fig. S3 LC-MS analysis of 20-prenylpaxilline and 22-prenylpaxilline. 9

Fig. S4 LC-MS analysis of 20-prenylpaxitriol and 22-prenylpaxitriol. 10

Fig. S5 LC-MS analysis of prenylated terpendole I formed by *in vivo* assay. 11

Fig. S6 HPLC profiles of feeding experiments to AO-*terF* with paxilline (**1**). 12

Fig. S7 SDS-PAGE of the heterogeneously expressed proteins. 13

Fig. S8 HPLC profiles of the prenylated β-paxitriol formed by *in vitro* assay. 14

Fig. S9 ^{1}H-NMR of 20-prenylpaxilline. 15

Fig. S10 ^{13}C-NMR of 20-prenylpaxilline. 16

Fig. S11 H-H COSY of 20-prenylpaxilline. 17

Fig. S12 HSQC of 20-prenylpaxilline. 18

Fig. S13 HMBC of 20-prenylpaxilline. 19

Fig. S14 NOESY of 20-prenylpaxilline. 20

Fig. S15 ^{1}H-NMR of 22-prenylpaxilline. 21

Fig. S16 ^{13}C-NMR of 22-prenylpaxilline. 22

Fig. S17 H-H COSY of 22-prenylpaxilline. 23

Fig. S18 HSQC of 22-prenylpaxilline. 24

Fig. S19 HMBC of 22-prenylpaxilline. 25

Fig. S20 NOESY of 22-prenylpaxilline. 26

Fig. S21 ^{1}H-NMR of β-paxitriol. 27

Fig. S22 ^{1}H-NMR of 20-prenylpaxitriol. 28

Fig. S23 ^{1}H-NMR of 22-prenylpaxitriol. 29

Reference 30

Supplementary experimental section

Compound identification based on GNPS analysis

Solid fermentation of *T. inflatum* was carried out in a 500 mL Fernbach flasks containing 80 g of rice and incubated at 25°C for 14 days. The fermented rice substrate was extracted with ethyl acetate, and the organic solvent was evaporated to dryness under vacuum to afford the crude extract. Crude extracts were subjected to UPLC-ESI-HRMS measurements (AB SCEIX Triple TOF 6600) with IDA acquisition modes. MS data preprocessing for feature-based molecular networking was performed using MZmine 2.53. MS data were analyzed via classical or feature-based molecular networking workflows, both of which are available in the GNPS web platform (https://gnps.ucsd.edu). The network file based on positive ion mode MS data can be found and accessed at https://gnps.ucsd.edu/ProteoSAFe/status.jsp?task=5af9d8f55b1449c08dda185ea9c581c9. Cytoscape V3.10.1 was used for visualization of relationships among compounds with yfileds circle layout.

Synthesis of β-paxitriol

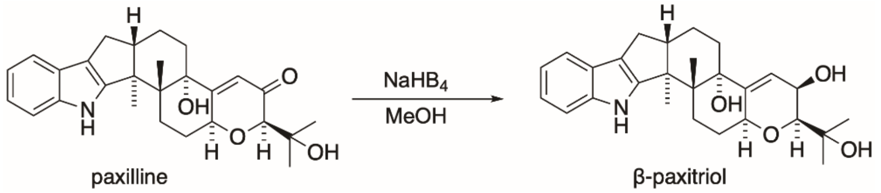

To a solution of paxilline (100.0 mg, 9.2 mmol) in MeOH (25 mL) was added $NaBH_4$ (52 mg, 55.0 mmol) and the mixture was stirred for 30 min at ambient temperature. The mixture was quenched by the addition of 1 mol NH_4Cl solution 12 ml .The mixture was added 1 mol HCl solution 25 ml and stirred for 30 min at ambient temperature. The aqueous layer was extracted with ethyl acetate , and the combined organic layers were washed with $NaHCO_3$, brine, dried over anhydrous $MgSO_4$, and concentrated in vacuo. Purification of the residue by preparative liquid chromatograph to gave β-paxitriol (10.0 mg, 914.8 μmol, 10%). The ^{1}H NMR data (Fig. S21) are in good agreement with the reported data (Miles et al. 1992).

Table S1: ^{1}H and ^{13}C NMR data of the compounds 20-prenylpaxilline (**5**) and 22-prenylpaxilline (**10**).

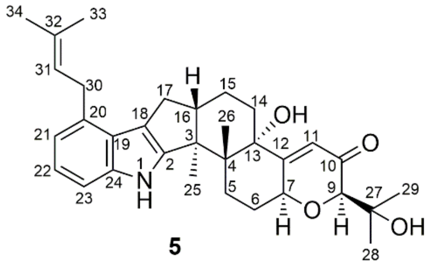

5

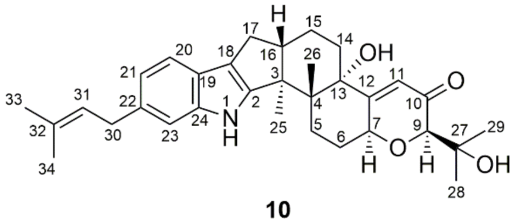

10

	5		10	
position	H-1	C-13	H-1	C-13
1	7.73 (1H, s)	-NH	7.64 (1H, s)	-NH
2	/	151.0	/	151.2
3	/	50.6	/	50.9
4	/	43.3	/	43.3
5	2.80 (1H, td, *J* = 13.6, 5.0 Hz)<br>1.47 (1H, dd, *J* = 13.2, 4.0 Hz)	28.2	2.82 (1H, m)<br>1.43 (1H, m)	28.1
6	2.33 (1H, m)<br>1.80 (1H, m)	28.6	2.33 (1H, m)<br>1.90 (1H, m)	28.6
7	4.86 (1H, m)	72.7	4.85 (1H, t, *J* = 9.0 Hz)	72.7
8	/	/	/	/
9	3.73 (1H, d, *J* = 2.0 Hz)	83.4	3.71 (1H, d, *J* = 1.8 Hz)	83.3
10	/	199.4	/	199.4
11	5.89 (1H, d, *J* = 7.9 Hz)	119.8	5.87 (1H, d, *J* = 1.6 Hz)	119.6
12	/	168.3	/	168.4
13	/	77.7	/	77.6
14	2.05 (1H, m)<br>1.66 (1H, m)	34.5	2.05 (1H, m)<br>1.67 (1H, m)	34.4
15	2.05 (1H, m)<br>1.82 (1H, m)	21.0	2.05 (1H, m)<br>1.82 (1H, m)	21.0
16	2.89 (1H, m)	49.6	2.82 (1H, m)	49.5
17	2.89 (1H, m)<br>2.61 (1H, m)	29.2	2.74 (1H, m)<br>2.42 (1H, m)	27.4
18	/	117.1	/	117.4
19	/	124.5	/	123.3
20	/	133.2	7.35(1H, d, *J* = 8.0 Hz)	118.6
21	6.86 (1H, d, *J* = 7.1 Hz)	119.2	6.92(1H, d, *J* = 8.0 Hz)	120.9
22	7.02 (1H, t, *J* = 7.1Hz)	121.2	/	134.7
23	7.15 (1H, d, *J* = 7.1 Hz)	109.4	7.11(1H, s)	110.9
24	/	139.8	/	140.4
25	1.54 (3H, s)	16.3	1.62 (3H, s)	16.3
26	1.05 (3H, s)	19.8	1.02 (3H, s)	19.8
27	4.10 (1H, s)	72.6	4.10 (1H, s)	72.7
28	1.31 (3H, s)	24.3	1.31 (3H, s)	24.3
29	1.31 (3H, s)	26.7	1.31 (3H, s)	26.7
30	3.61 (2H, d, *J* = 7.0 Hz)	32.1	3.41 (2H, d, *J* = 7.2 Hz)	34.6
31	5.41 (1H, t, *J* = 7.0 Hz)	123.7	5.36 (1H, t, *J* = 7.2 Hz)	124.3
32	/	132.0	/	131.9
33	1.77 (3H, s)	18.1	1.74 (3H, s)	17.8
34	1.76 (3H, s)	25.9	1.74 (3H, s)	25.7

Table S2: DNA and protein sequences of *tolF*. Highlighted in yellow is the intron region.

Gene	DNA sequence	Protein sequence
TorF	ATGACTTTTGACAAGGAGGCGGTGACAGCTCCAGCACGCG<br>ATAGCAAACATGCTGCAGACCTGGAATACTGGACGCAACAT<br>GTTGTCCCTATTATCAGCTCCCTCCTAAAGTCTGCCGGATCC<br>TACTCGCCTGACGACCAGGACGCGCACATACGTACCCTATC<br>AGAACATGTCTTCCCGAACCTTGGTCCGCGGCCATCCATGG<br>CTCATACCAGGTCCTTTTTGACCCAGACCGGCTCCCCTCTCC<br>AGCCCAGCATCAACTTCAGCTCCGGAAAGCCCCAAGTACGT<br>TACTGCTGGGAGCTGCTGGGAGCTCAAGGCGGCAGCGATG<br>GCGACCCGCTTGCGGTGGAGGCAGCGCGAGAGATACTGTC<br>TTATCTTTCCACGGCCTTTGGCTTCAGCACACGATGGAGCG<br>ACGCCTGGCTGTCCGCATTTGCTCCAACACTAGAAGAAGCG<br>AAATCCGTCCAGGTCAAGCTCCCGAAGTGGCTGGCGAGCTT<br>CACGTCGGCGGAGGAAGAAGTGCCCGCGTTGAAGCGACTT<br>CCCTTTGCCTTTGTTGCCTTTGACTTGAGTGGCCCCAAGAC<br>GTCTATGAAGGCGTACTTTAATCCCAAGGGCAAGGAAATCG<br>CAACTGGGAAGCCAGCAGCGGATGCGACTTGGAGTACTCTT<br>CGCAGTCTGAAACCGTCTCTGAACACGGCGTCAATCGACAT<br>CCTTGAGCAGTGAGTCCGAGCTCGGCCTTGGCCATTTGATG<br>TTTCTTCAATGTTCAGACATTTGCTTACTTCGTCTGGAACAG<br>ATTCCTTGCCGAACGCGCAGTACCTTCCACGGTCGAGCTTG<br>TGGGAATCGACTGTGTCGACGAAGCCAGTCTGTCGGATGCA<br>AGGGTCAAGCTCTACGTCTATACCTTGAGCAACTCCTTCGA<br>AACGGTTCGCGACTACGTGACCCTGGGCGGCCGTCTCCAGG<br>ATGAGACTACCTTGAAGGGCTTGGACATCTTGCACGACATT<br>TGGCACCTCCTGCTCCAAGAGCCCGAAGGCGTCAACGACG<br>ACTACAACAAGCCCGTCAACGACGGTTCCATCCTCTGCCAG<br>AAGCTGTACTTTAGCTTCGAGATGAGGCCCGGCAGGGAGCT<br>TCCCGAAGTCAAGTCCTACGTACCGACTTGGAACTATGTAC<br>GGAGCGATGCGGAAACCGTTCAAAACTACGAGGAGGTCTT<br>CCGAAGATGCGGGTACGAGTGGGGAGAGGATGGGAGGTAC<br>AAGAAACTGTTCGAGAGTGCTTTGTGTGTTTCCCTCCCTAC<br>CATGATGTAAGCATTCACTAACCGTTCATTCTCAGCGGGCCG<br>GTGGAACACAACCGCCCGAAGCCGGTCCATTGCGACGCAT<br>CGTTCCTATACTCCGAAAAGAAGGGGACATATCAGACACTG<br>TACTACAGCCCACCGCTCGGAGAAGAAGAATACAAGTAG	MTFDKEAVTAPAR<br>DSKHAADLEYWT<br>QHVVPIISSLLKSA<br>GSYSPDDQDAHIR<br>TLSEHVFPNLGPRP<br>SMAHTRSFLTQTG<br>SPLQPSINFSSGKP<br>QVRYCWELLGAQ<br>GGSDGDPLAVEAA<br>REILSYLSTAFGFS<br>TRWSDAWLSAFAP<br>TLEEAKSVQVKLP<br>KWLASFTSAEEEV<br>PALKRLPFAFVAFD<br>LSGPKTSMKAYFN<br>PKGKEIATGKPAA<br>DATSTLRSLKPSLN<br>TASIDILEQFLAER<br>AVPSTVELVGIDCV<br>DEASLSDARVKLY<br>VYTLSNSFETVRD<br>YVTLGGRLQDETT<br>LKGLDILHDIWHL<br>LLQEPEGVNDDYN<br>KPVNDGSILCQKL<br>YFSFEMRPGRELP<br>EVKSYVPTWNYV<br>RSDAETVQNYEEV<br>FRRCGYEWGEDG<br>RYKKLFESAFGPV<br>EHNRPKPVHCDAS<br>FLYSEKKGTYQTL<br>YYSPPLGEEEYK

Table S3: Plasmids used in this study.

Primer	Sequence 5'-3'	Restriction site	Vector
TolF-F1	ccggaattcgagctcggtaccATGACTTTTGACAAGGAGGCGGTG	*KpnI*	pUARA2
TolF-R1	tactacagatccccgggtaccCTACTTGTATTCTTCTTCTCCGAG		
TolF-F2	ctcggtaccctcgagggatccATGACTTTTGACAAGGAGGCGGTG	*BamHI*	pCold
TolF-R2	gacaagcttgaattcggatccCTACTTGTATTCTTCTTCTCCGAG		
TolF-F3	caccatcaccatcacggatccATGACTTTTGACAAGGAGGCGGTG	*BamHI*	pQE30
TolF-R3	accgagctcgcatgcggatccCTACTTGTATTCTTCTTCTCCGAG		
TolF-F4	cagcaaatgggtcgcggatccATGACTTTTGACAAGGAGGCGGTG	*BamHI*	pET28a
TolF-R4	acggagctcgaattcggatccCTACTTGTATTCTTCTTCTCCGAG		
TolF-F5	cgcgatatcgtcgacggatccATGACTTTTGACAAGGAGGCGGTG	*BamHI*	pMal-c5x
TolF-R5	acctgcagggaattcggatccCTACTTGTATTCTTCTTCTCCGAG		
TerF-F1	ccggaattcgagctcggtaccATGACTATCGACAAGAAGCCGGTG	*KpnI*	pUARA2
TerF-R1	tactacagatccccgggtaccCTATTCGTACTCTTCGCCATCTTTC		
TerF-F2	cgcgatatcgtcgacggatccATGACTATCGACAAGAAGCCGGTG	*BamHI*	pMal-c5x
TerF-R2	acctgcagggaattcggatccCTATTCGTACTCTTCGCCATCTTTC		

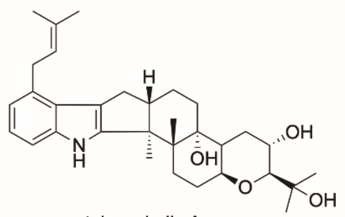

tolypocladin A

MW: 507.72

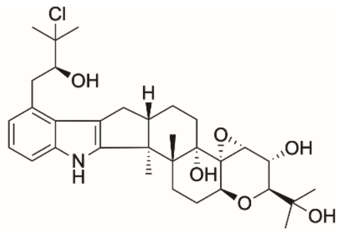

tolypocladin B

MW: 574.16

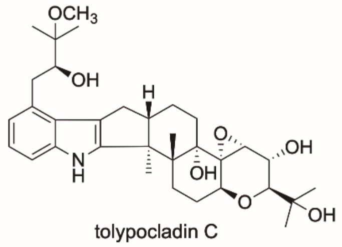

tolypocladin C

MW: 569.74

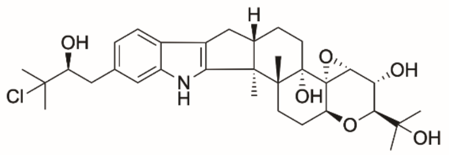

tolypocladin D

MW: 574.16

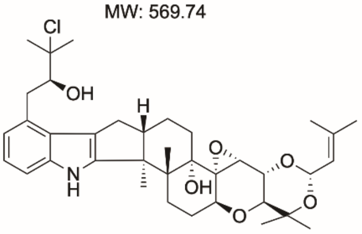

tolypocladin E

MW: 640.26

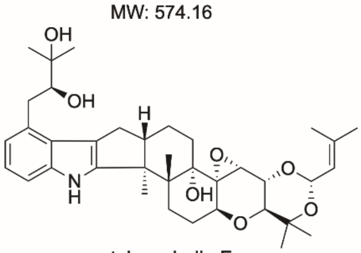

tolypocladin F

MW: 621.82

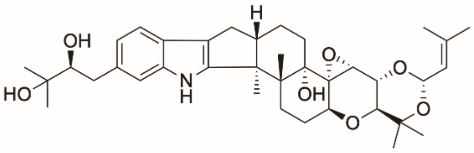

tolypocladin G

MW: 621.82

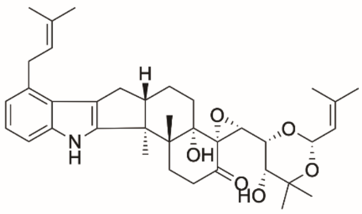

tolypocladin H

MW: 603.80

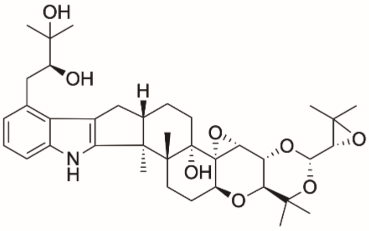

tolypocladin I

MW: 637.8140

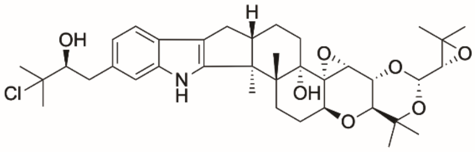

tolypocladin J

MW: 656.2570

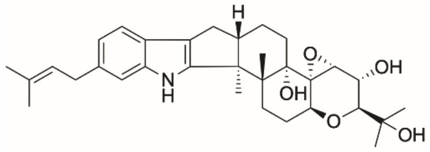

tolypocladin K

MW: 521.70

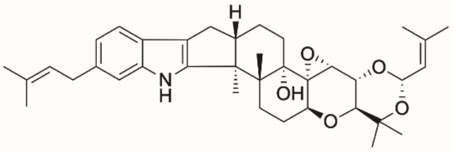

tolypocladin L

MW: 587.80

Fig. S1 Chemical structure of tolypocladin A-L (Xu et al. 2019a; Xu et al. 2019b).

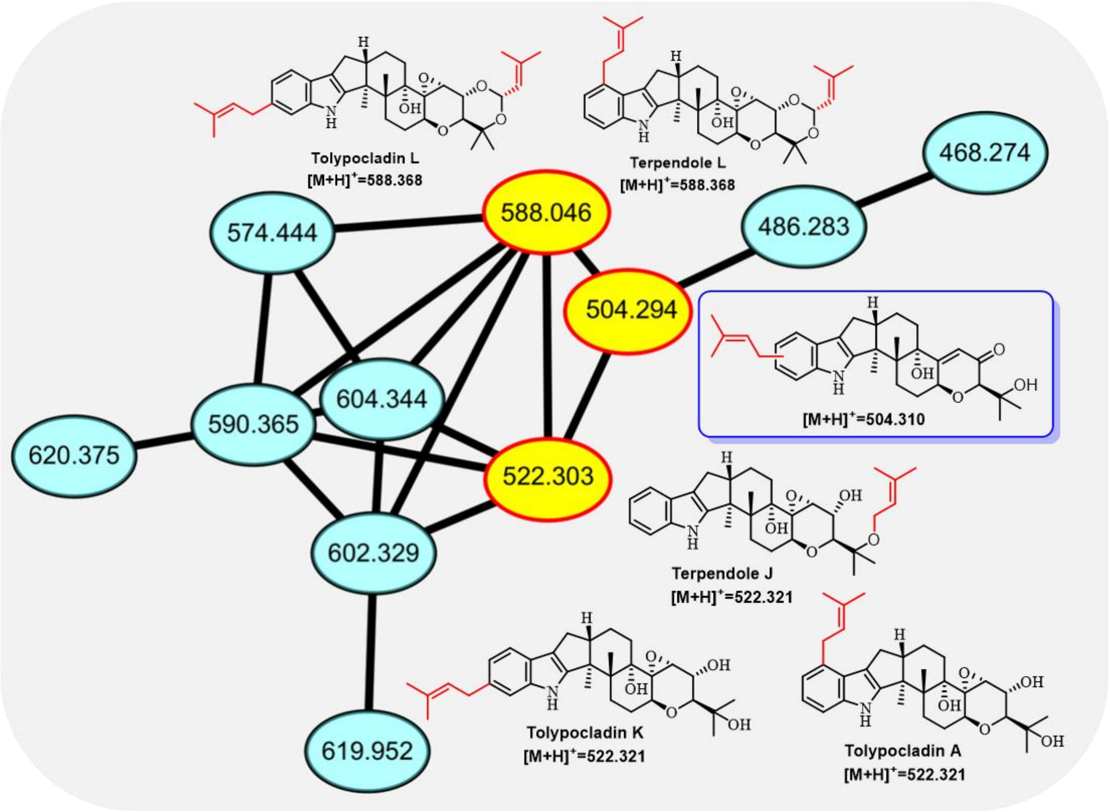

Fig. S2 Molecular network of the metabolic products from *T. inflatum*.

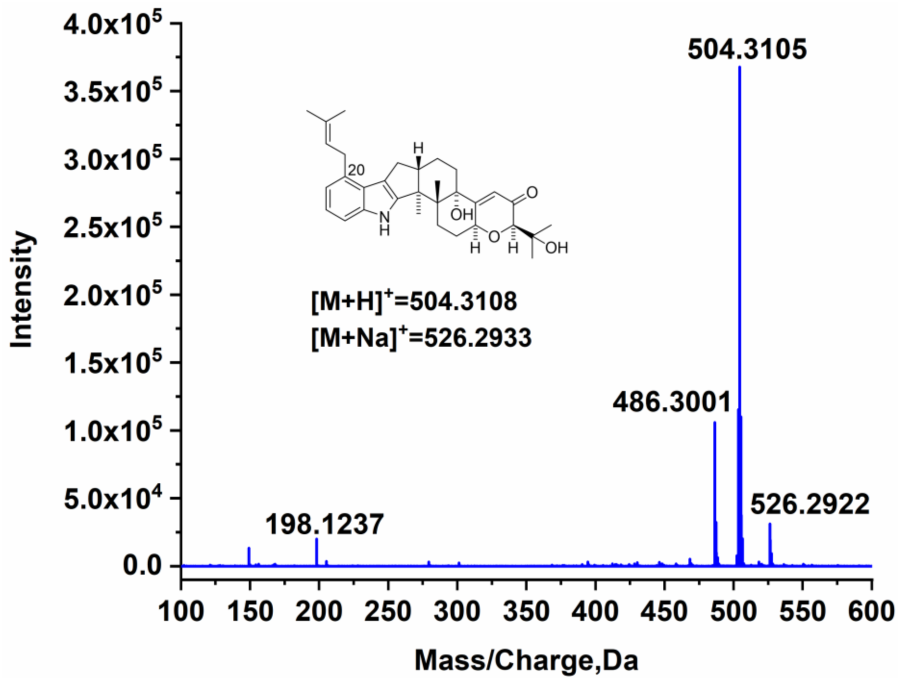

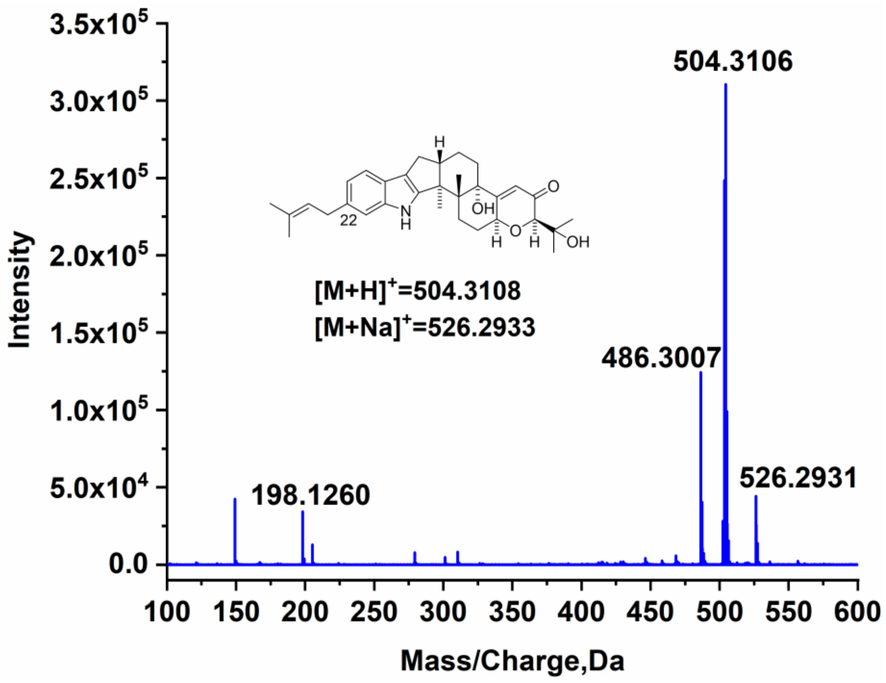

Fig. S3 LC-MS analysis of 20-prenylpaxilline and 22-prenylpaxilline.

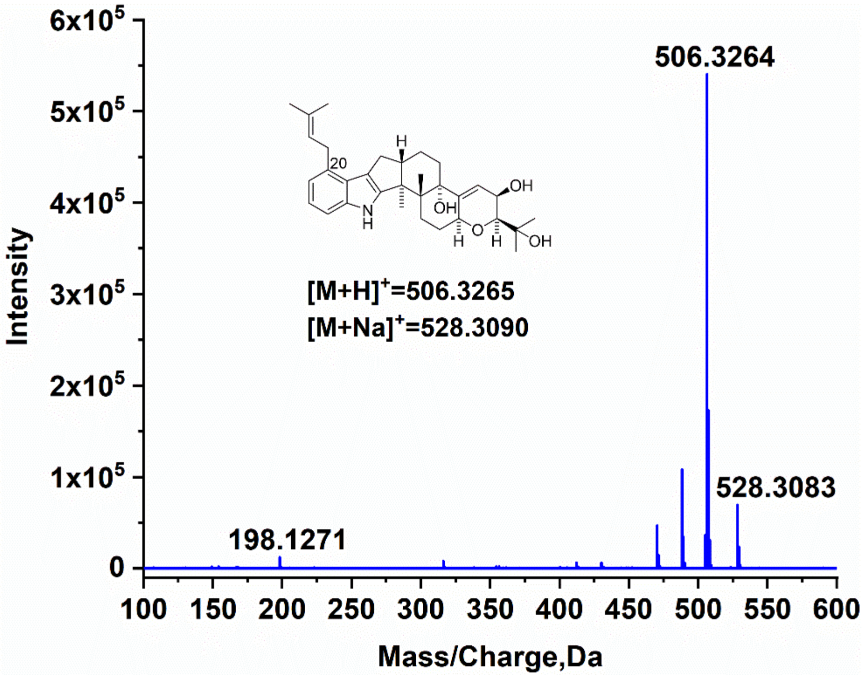

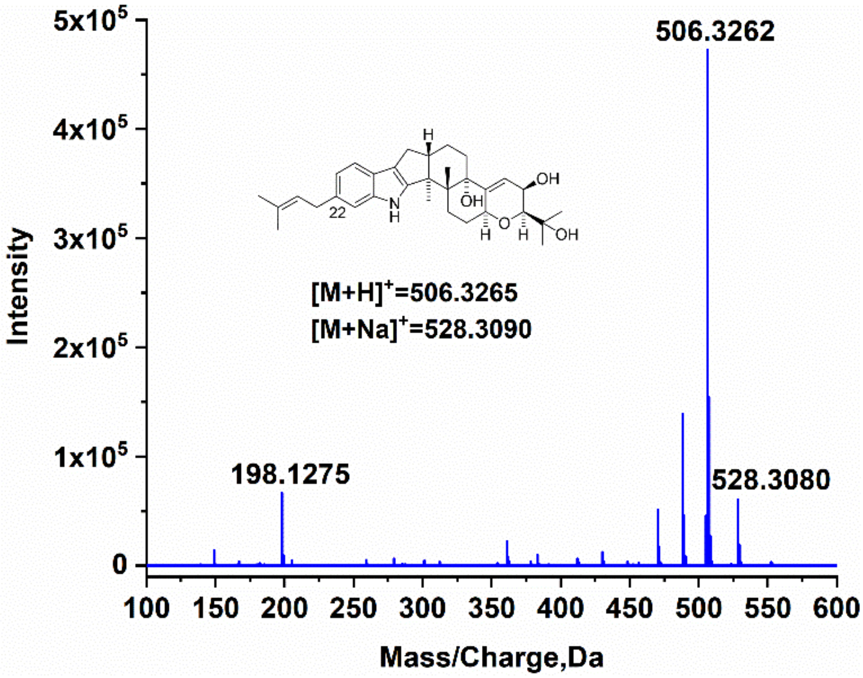

Fig. S4 LC-MS analysis of 20-prenylpaxitriol and 22-prenylpaxitriol.

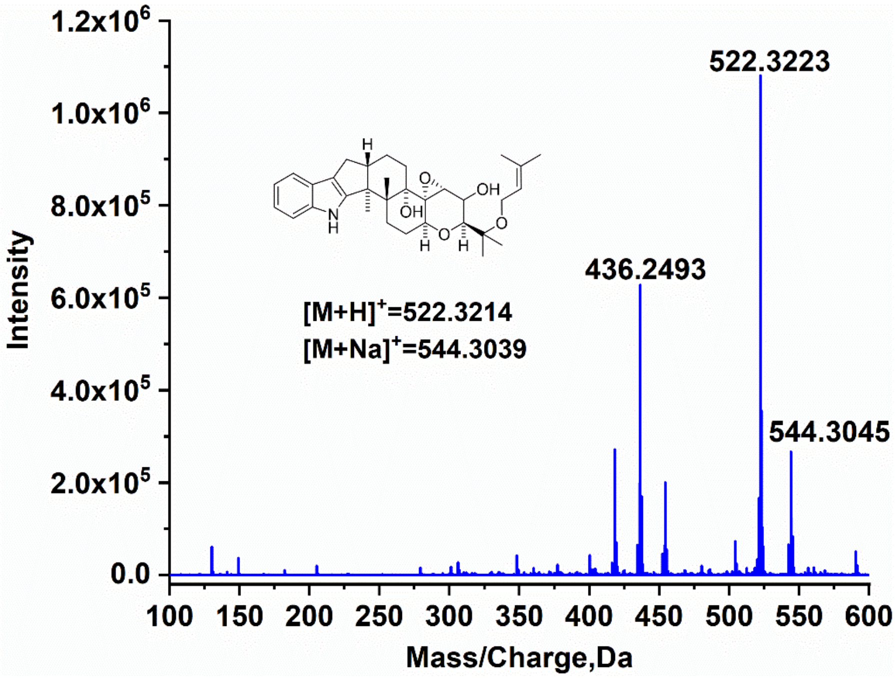

Fig. S5 LC-MS analysis of prenylated terpendole I formed by *in vivo* assay.

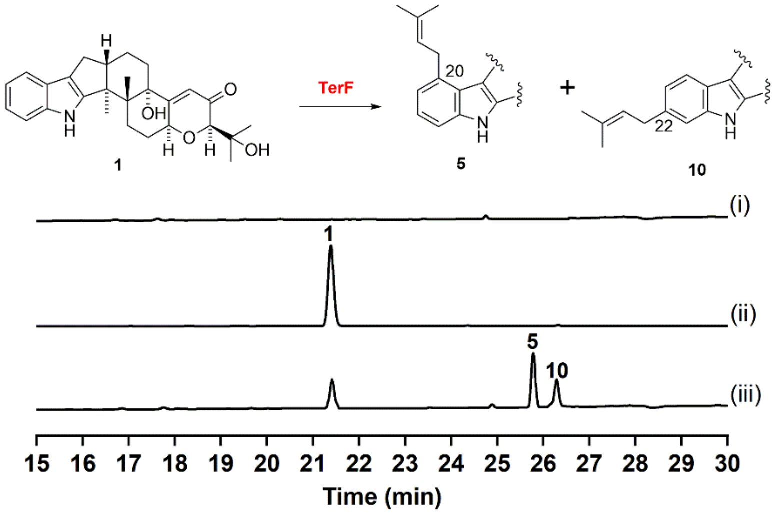

Fig. S6 HPLC profiles of feeding experiments to *AO-terF* with paxilline (**1**). (i) *AO-WT*; (ii) *AO-WT* + **1**; (iii) *AO-terF* + **1**.

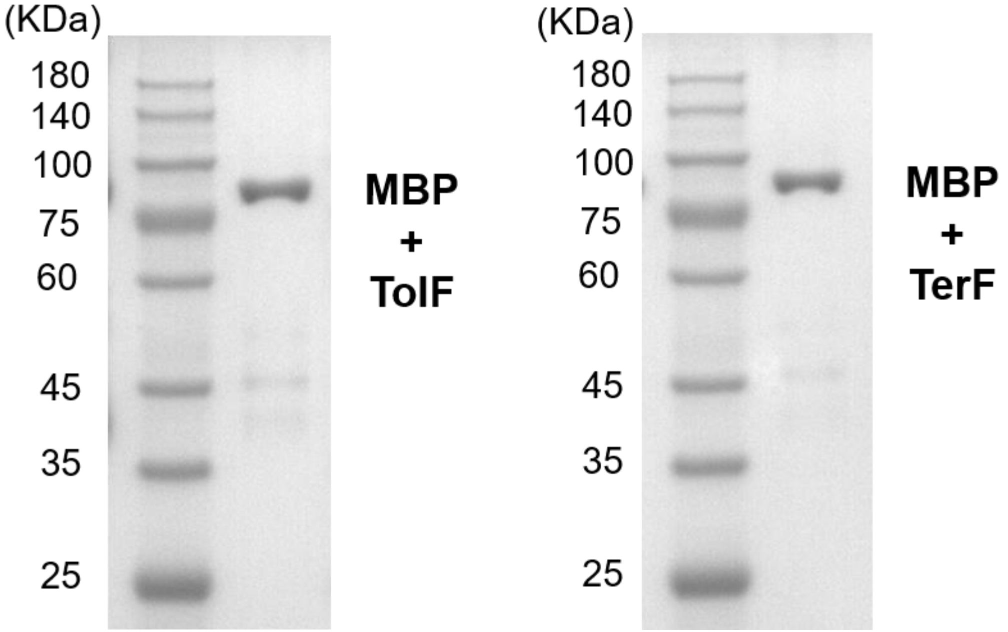

Fig. S7 SDS-PAGE of the heterogeneously expressed proteins. TolF ≈ 47 KDa + MBP tag = 42.5 KDa (~89.5 KDa). TerF≈ 46.2 KDa + MBP tag = 42.5 KDa (~88.8 KDa).

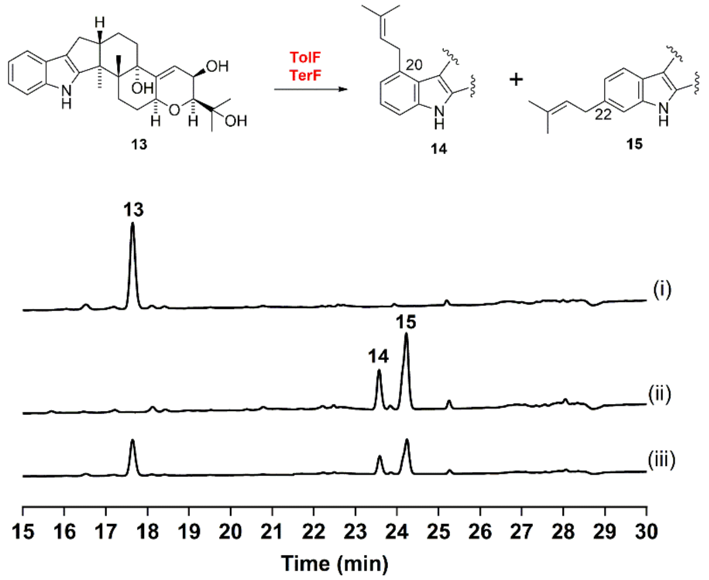

Fig. S8 HPLC profiles of the prenylated β-paxitriol formed by in vitro assay. (i) boiled enzyme with **13** and DMAPP; (ii) TolF with **13** and DMAPP; (iii) TerF with **13** and DMAPP.

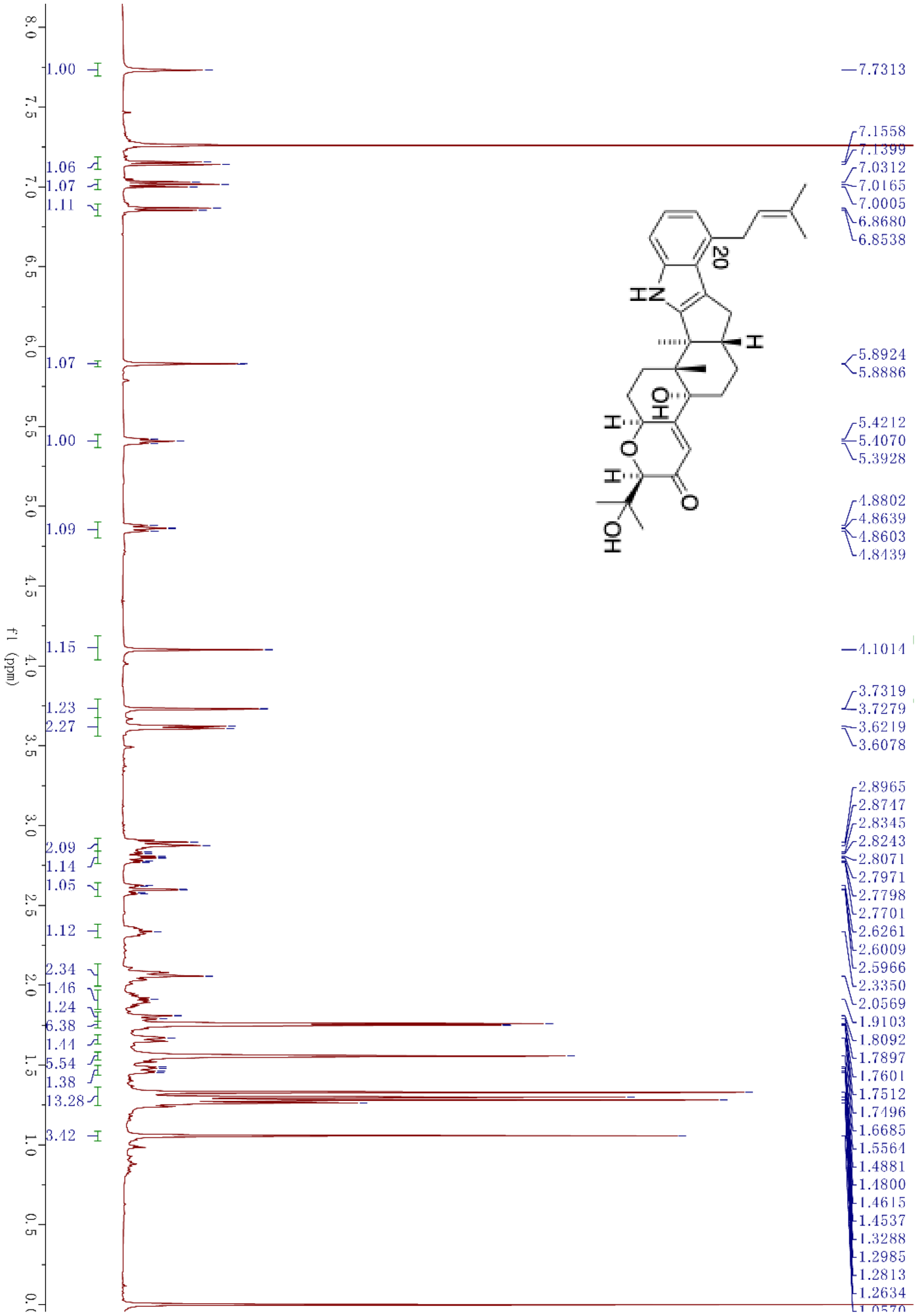

Fig. S9 ^{1}H-NMR of 20-prenylpaxilline.

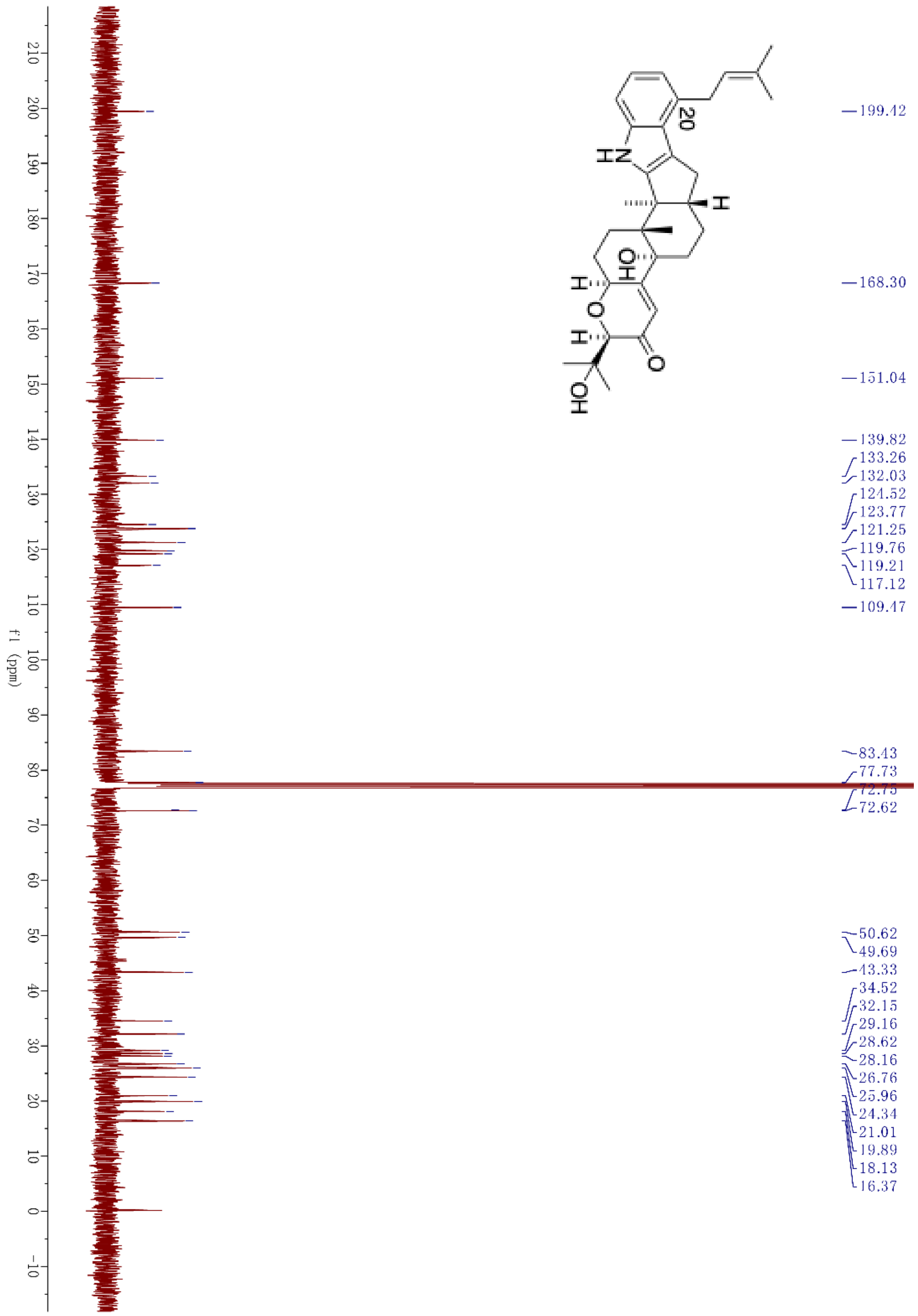

Fig. S10 ^{13}C-NMR of 20-prenylpaxilline.

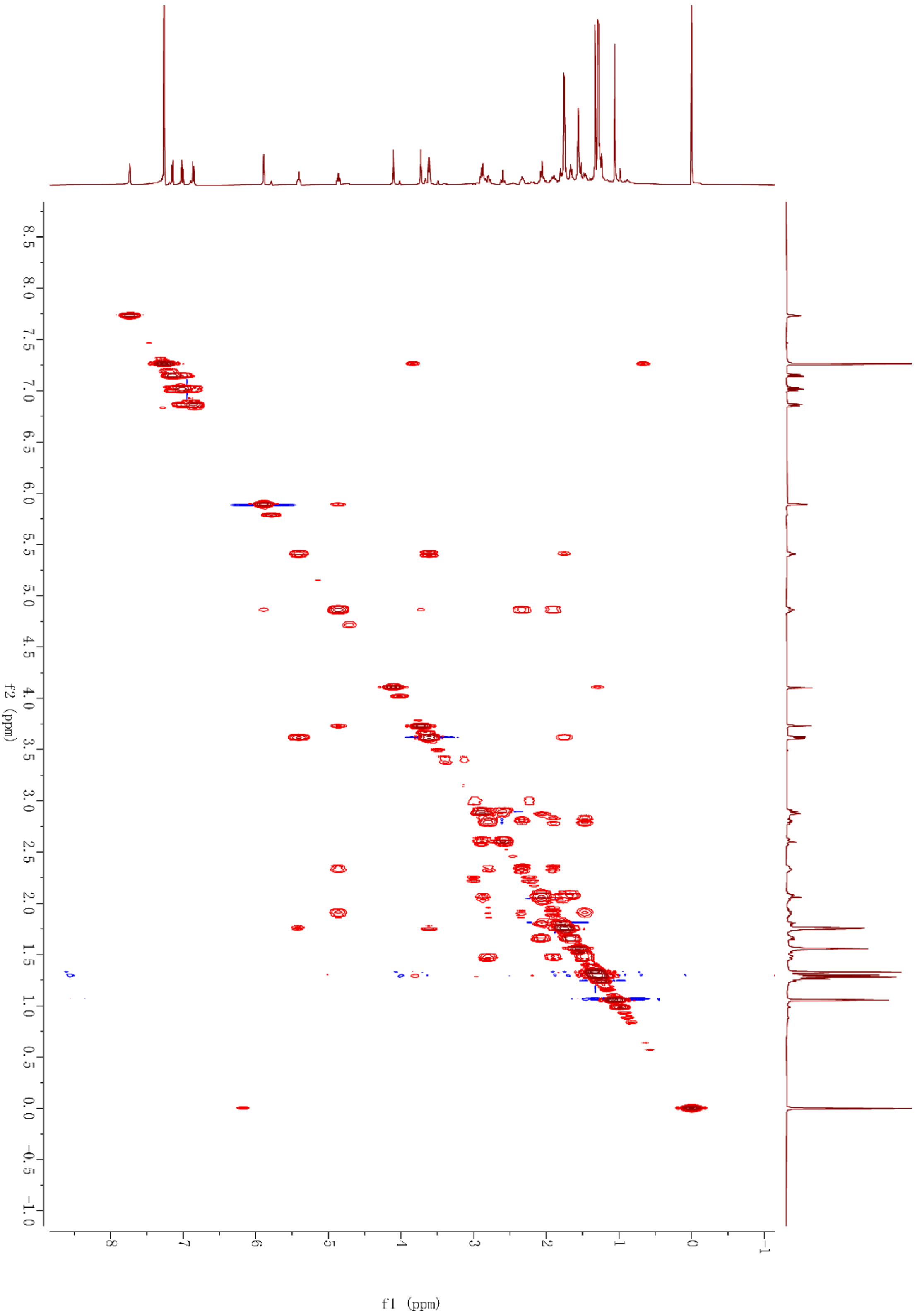

Fig. S11 H-H COSY of 20-prenylpaxilline.

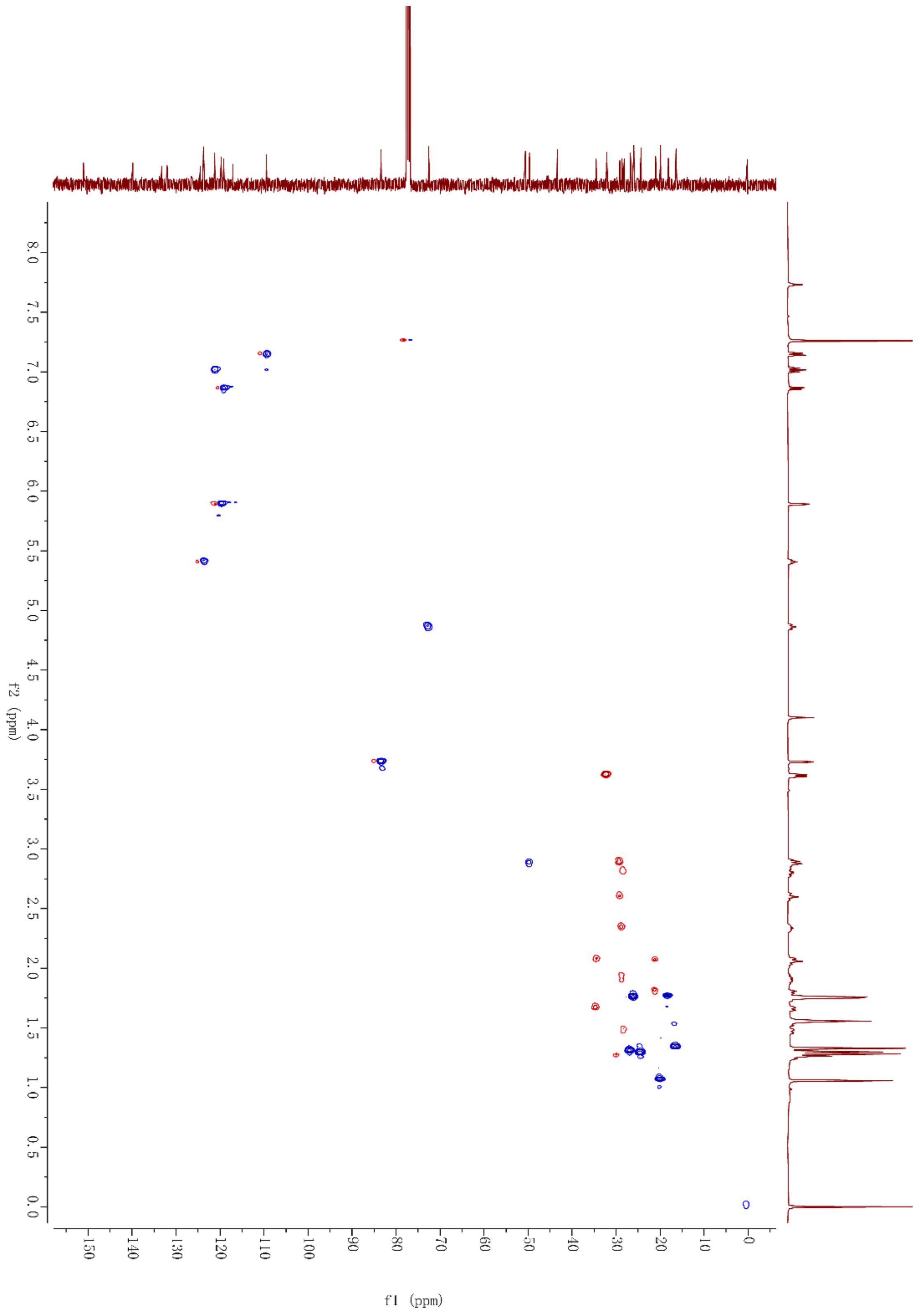

Fig. S12 HSQC of 20-prenylpaxilline.

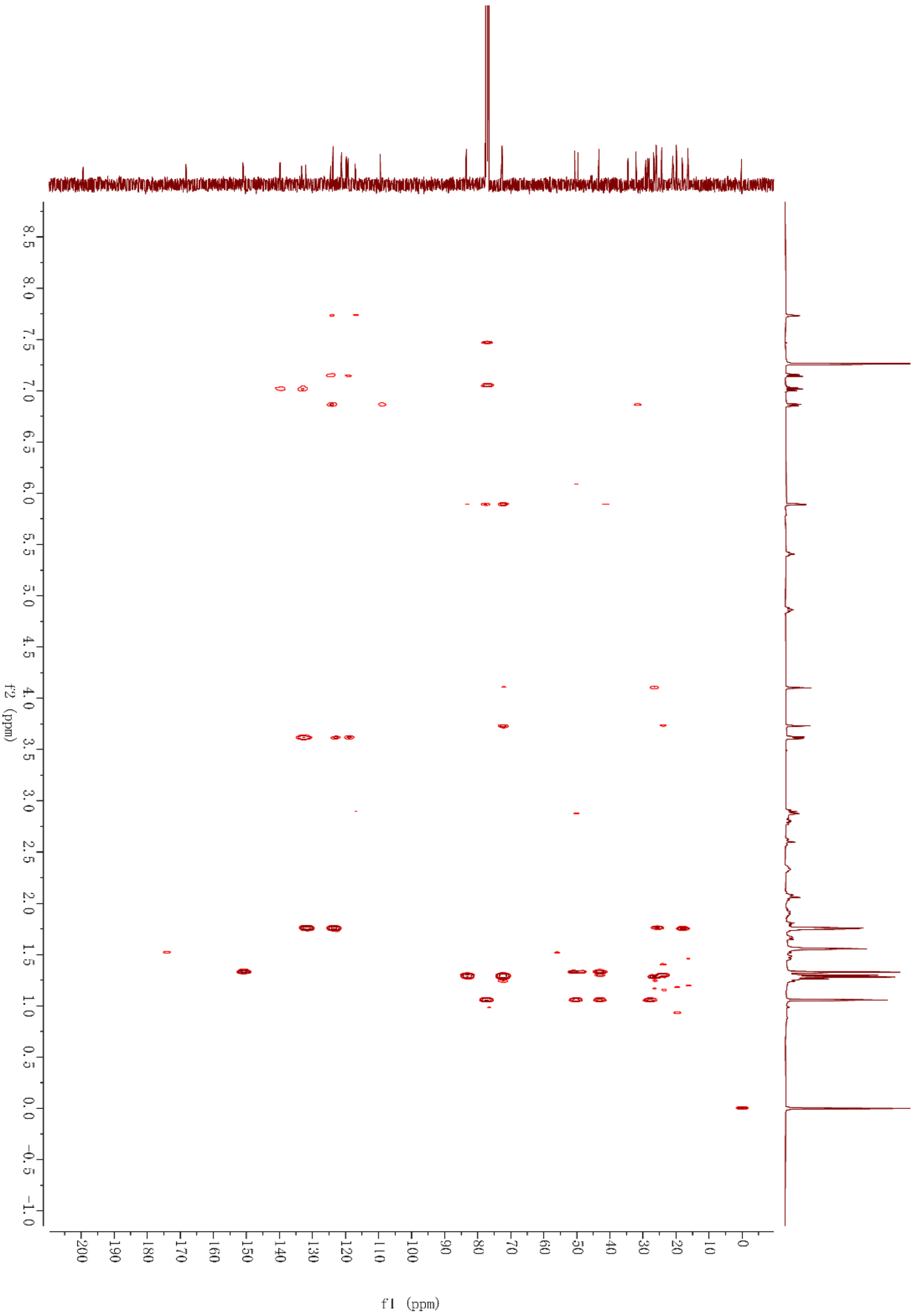

Fig. S13 HMBC of 20-prenylpaxilline.

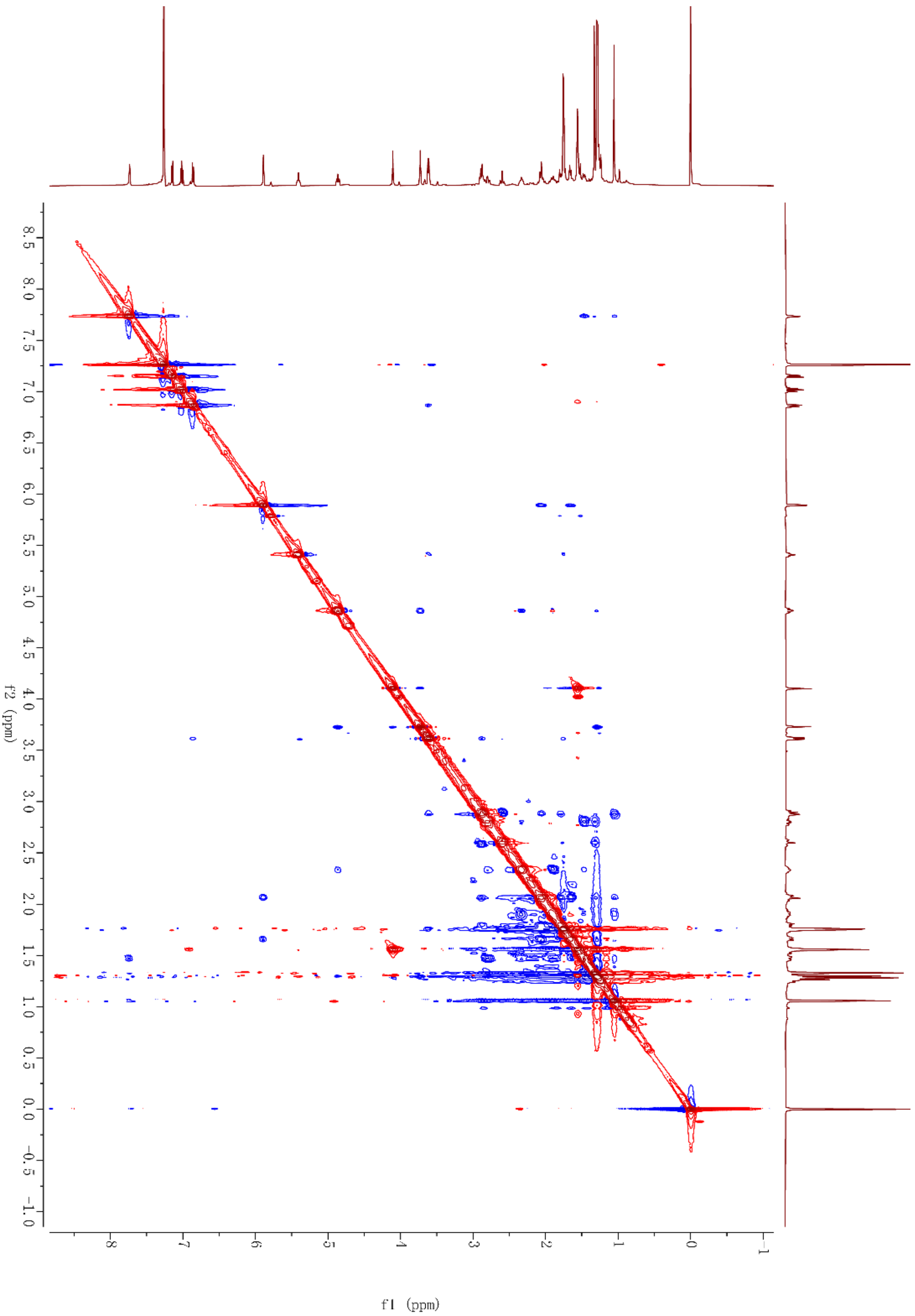

Fig. S14 NOESY of 20-prenylpaxilline.

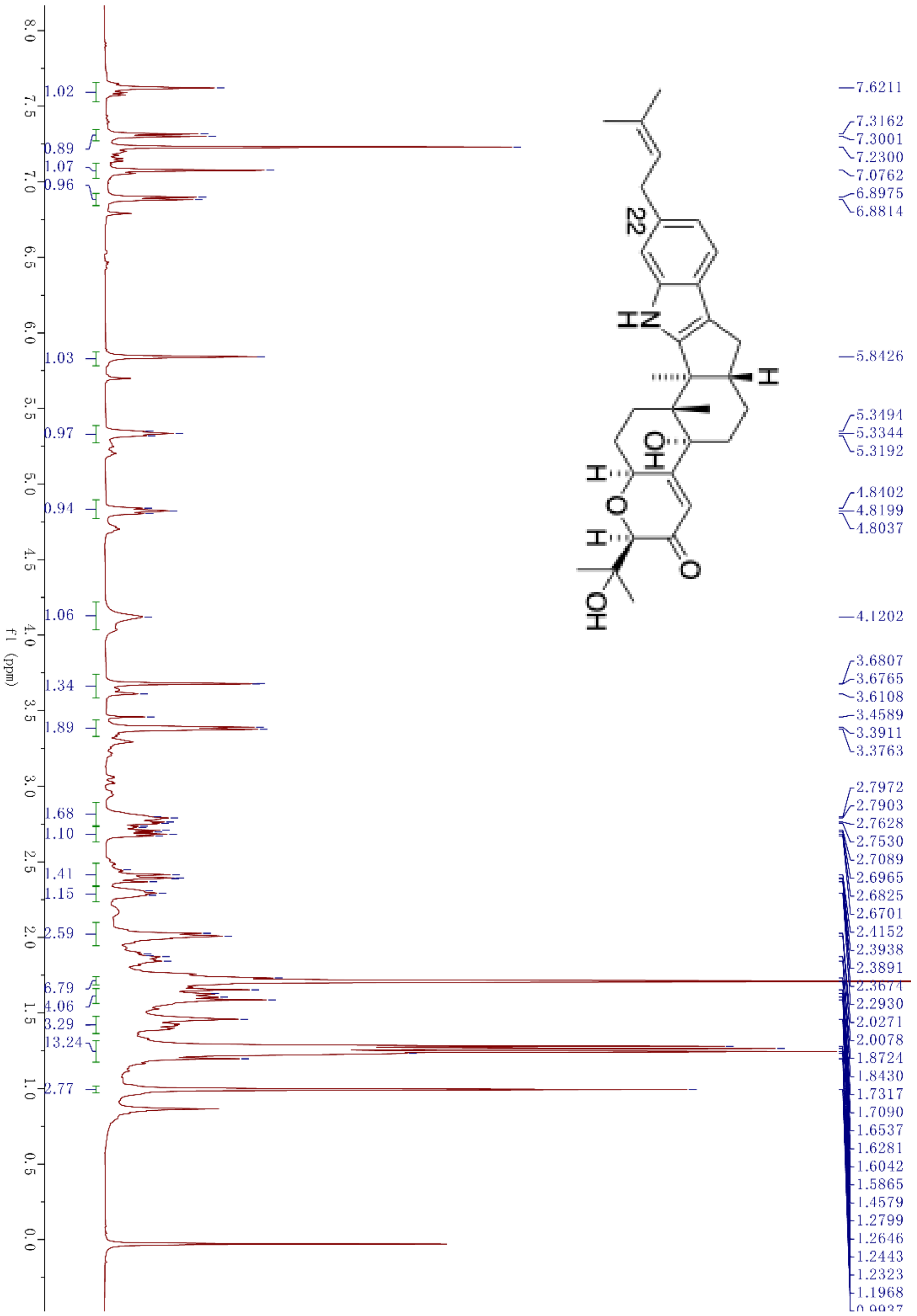

Fig. S15 ^{1}H-NMR of 22-prenylpaxilline.

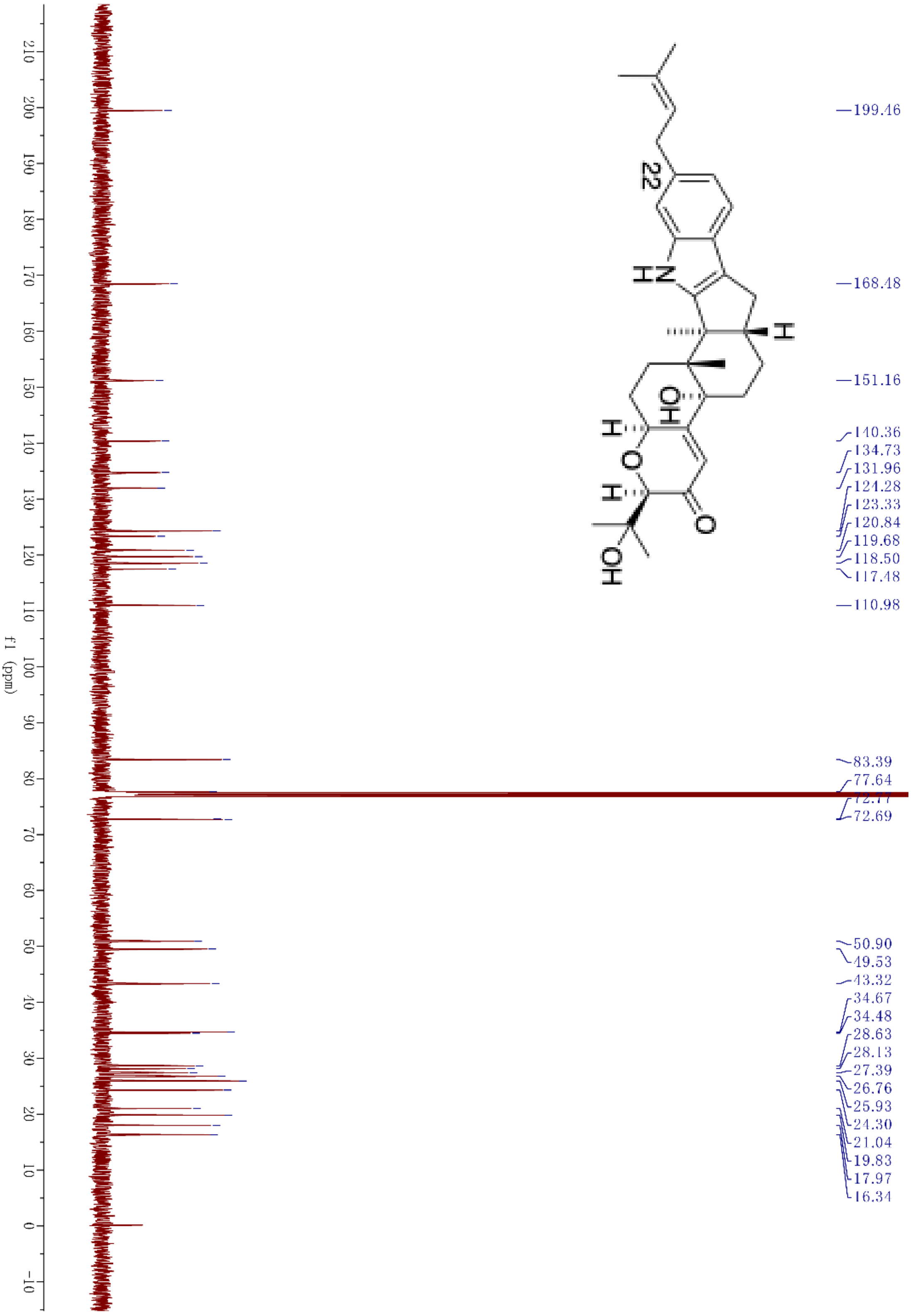

Fig. S16 ^{13}C-NMR of 22-prenylpaxilline.

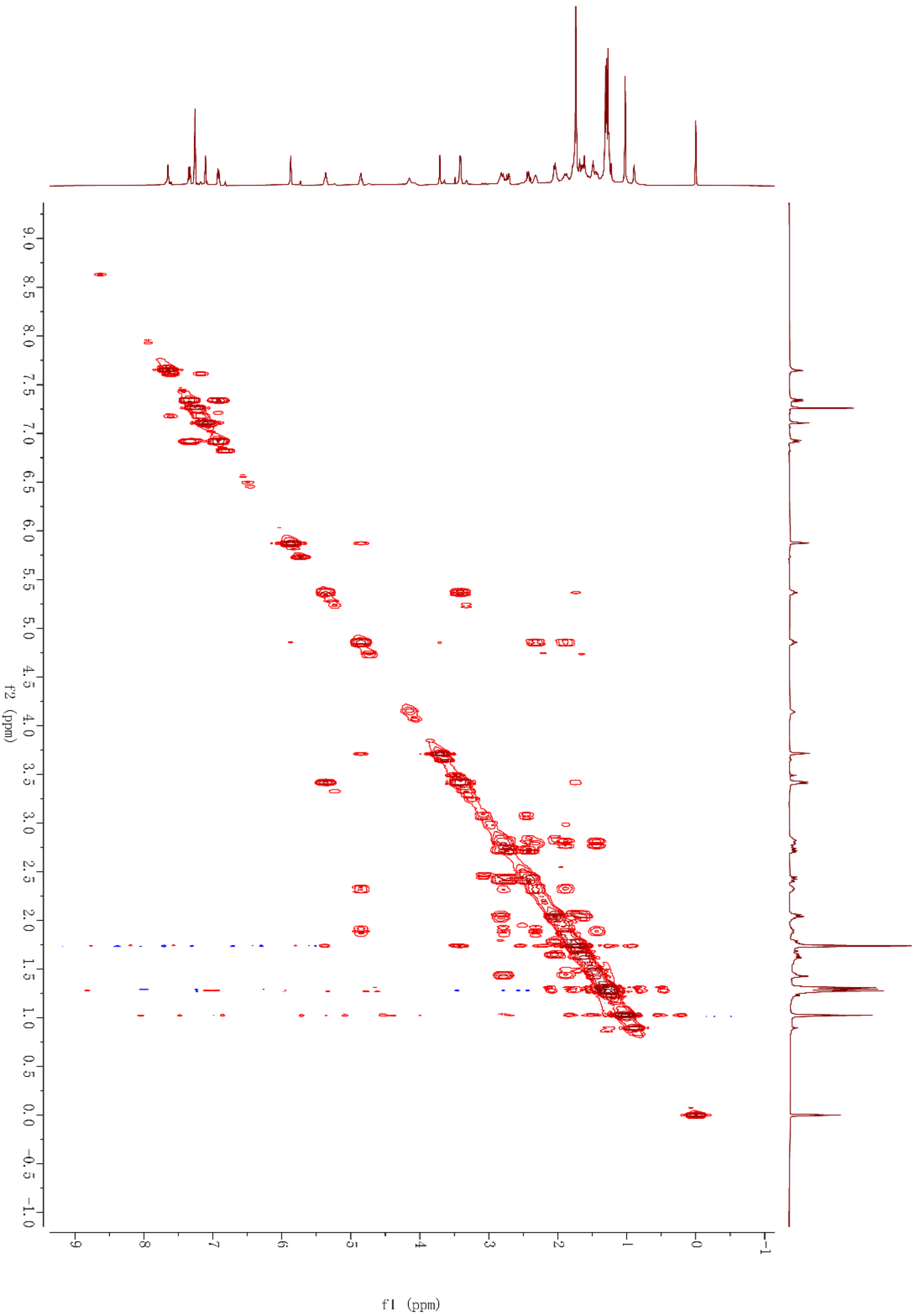

Fig. S17 H-H COSY of 22-prenylpaxilline.

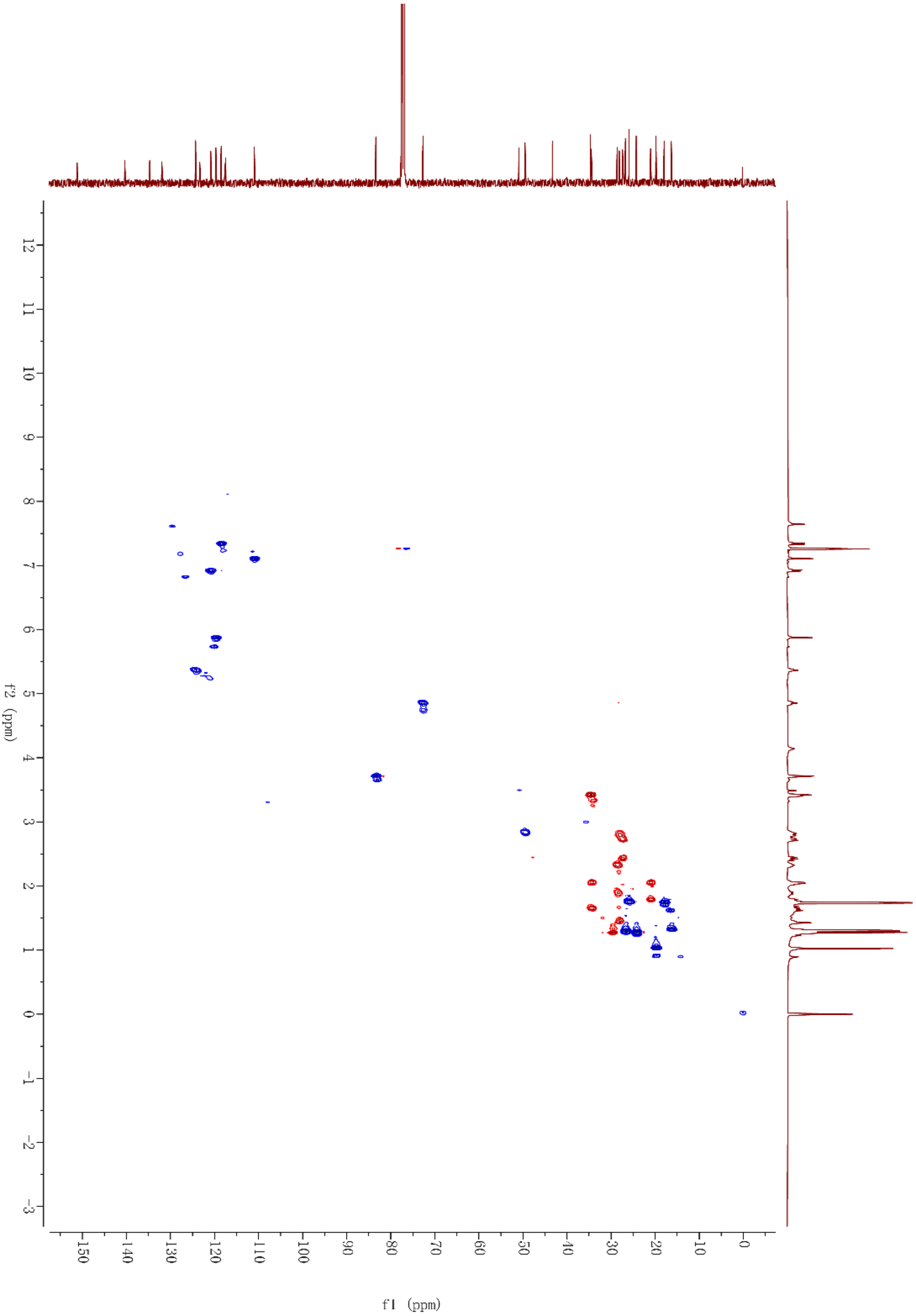

Fig. S18 HSQC of 22-prenylpaxilline.

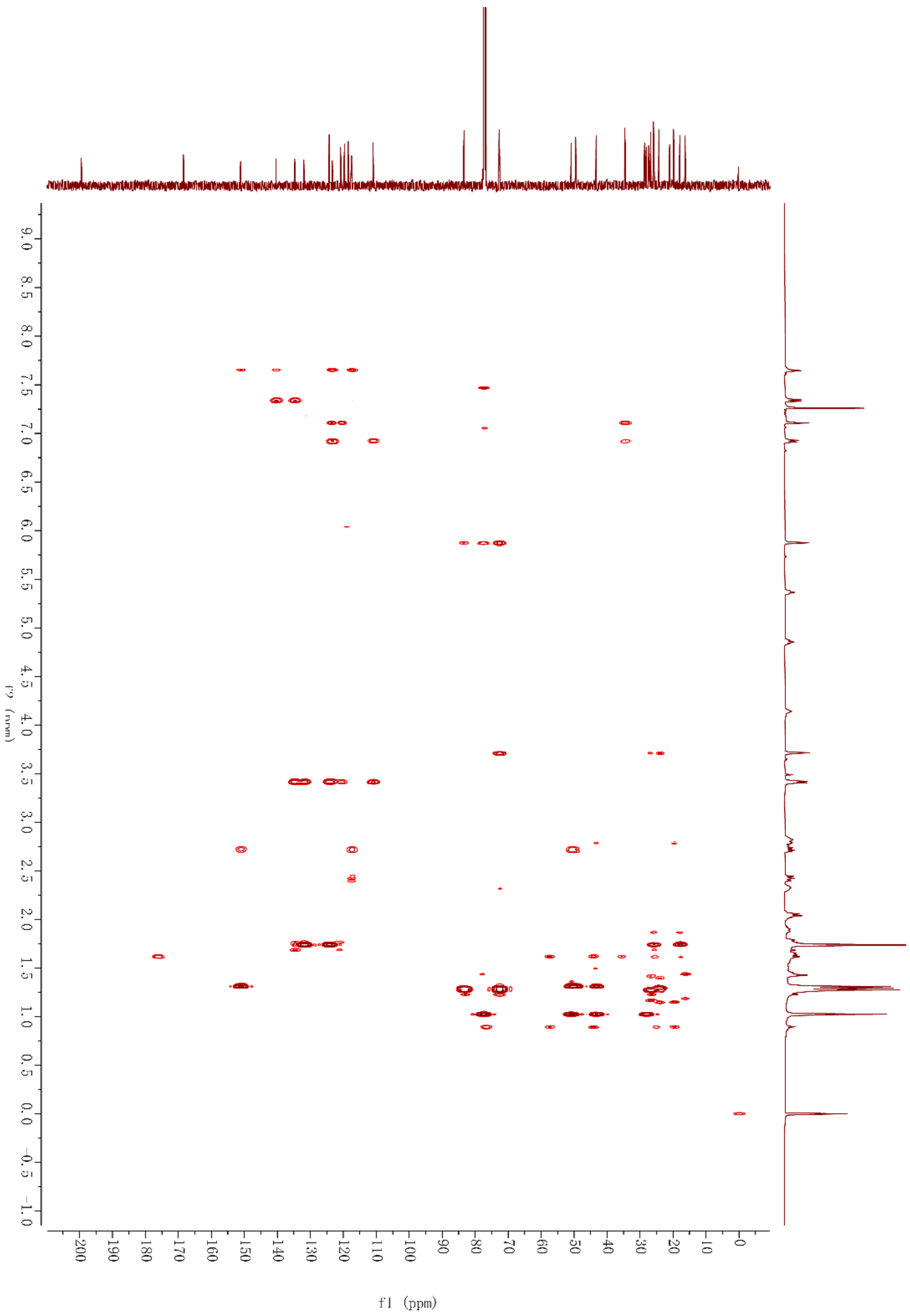

Fig. S19 HMBC of 22-prenylpaxilline.

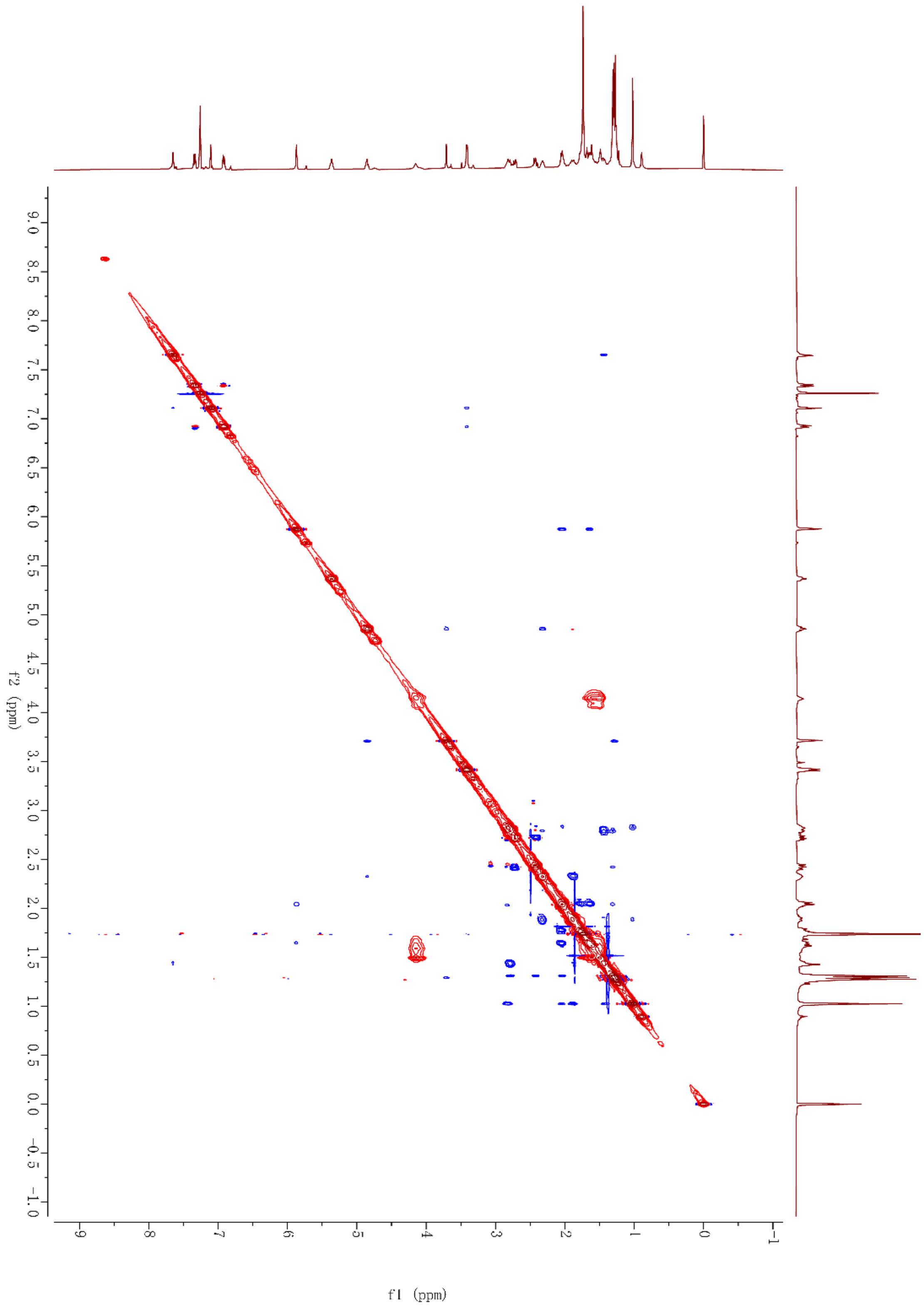

Fig. S20 NOESY of 22-prenylpaxilline.

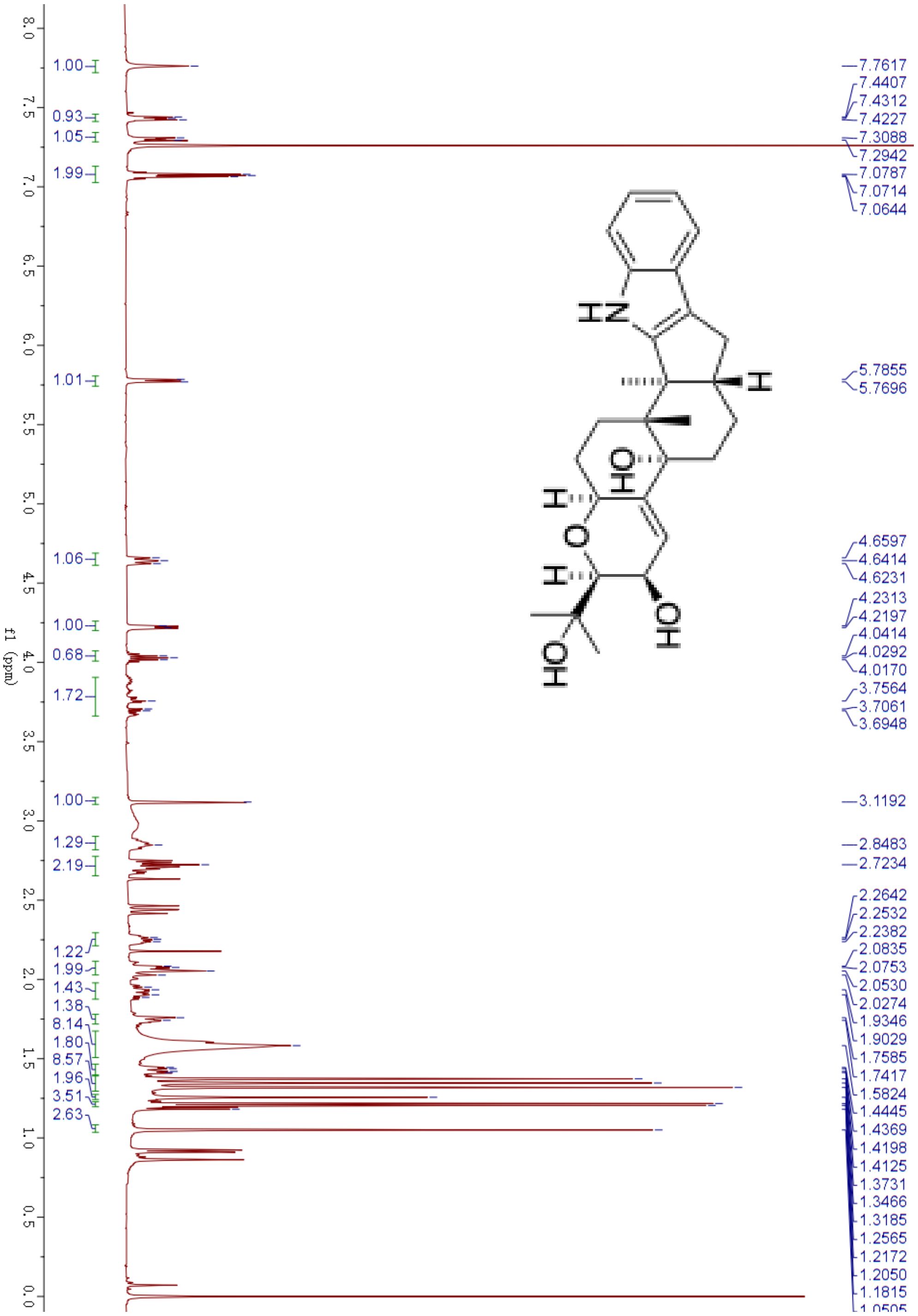

Fig. S21 ^{1}H-NMR of β-paxitriol.

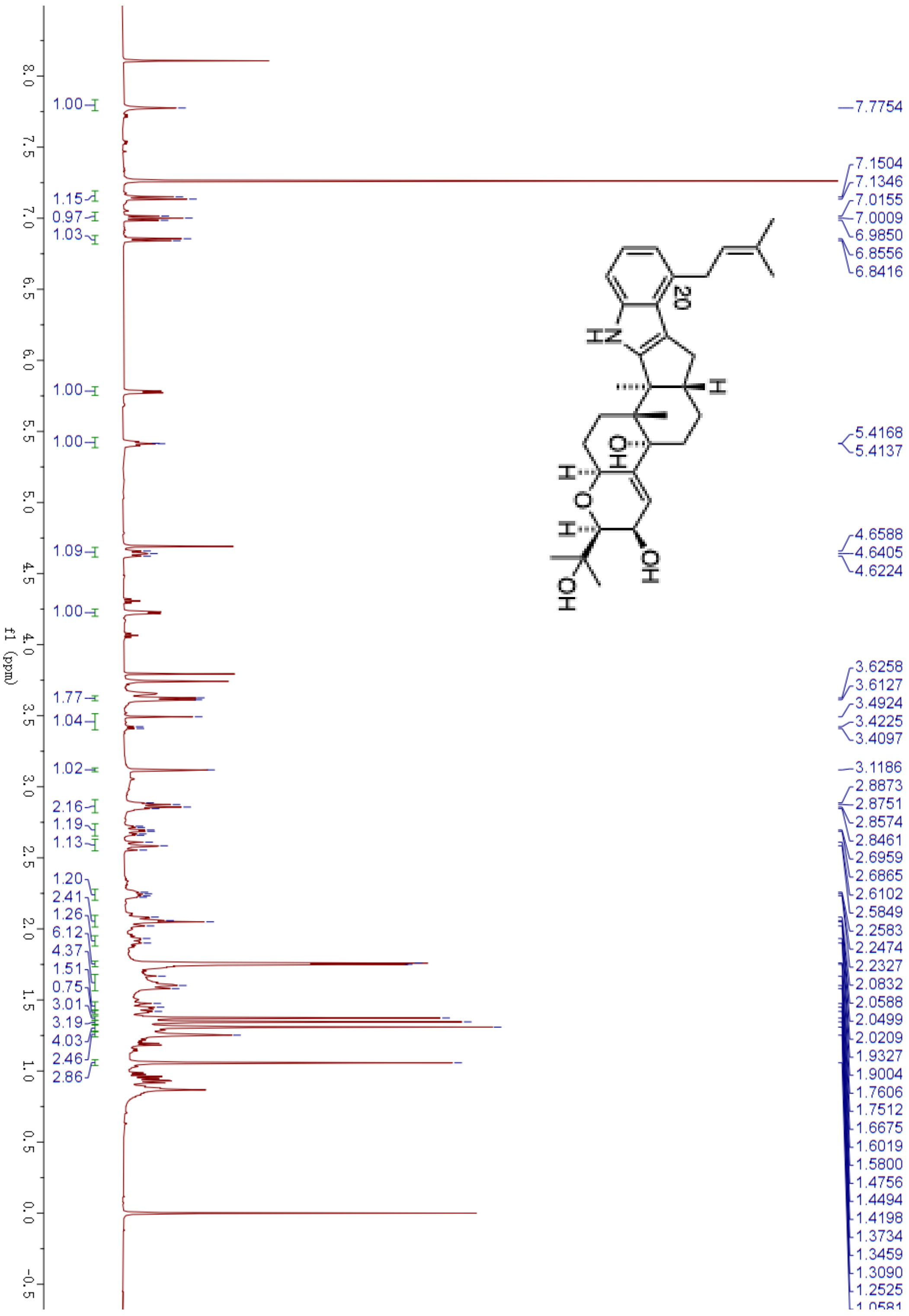

Fig. S22 ^{1}H-NMR of 20-prenylpaxitriol.

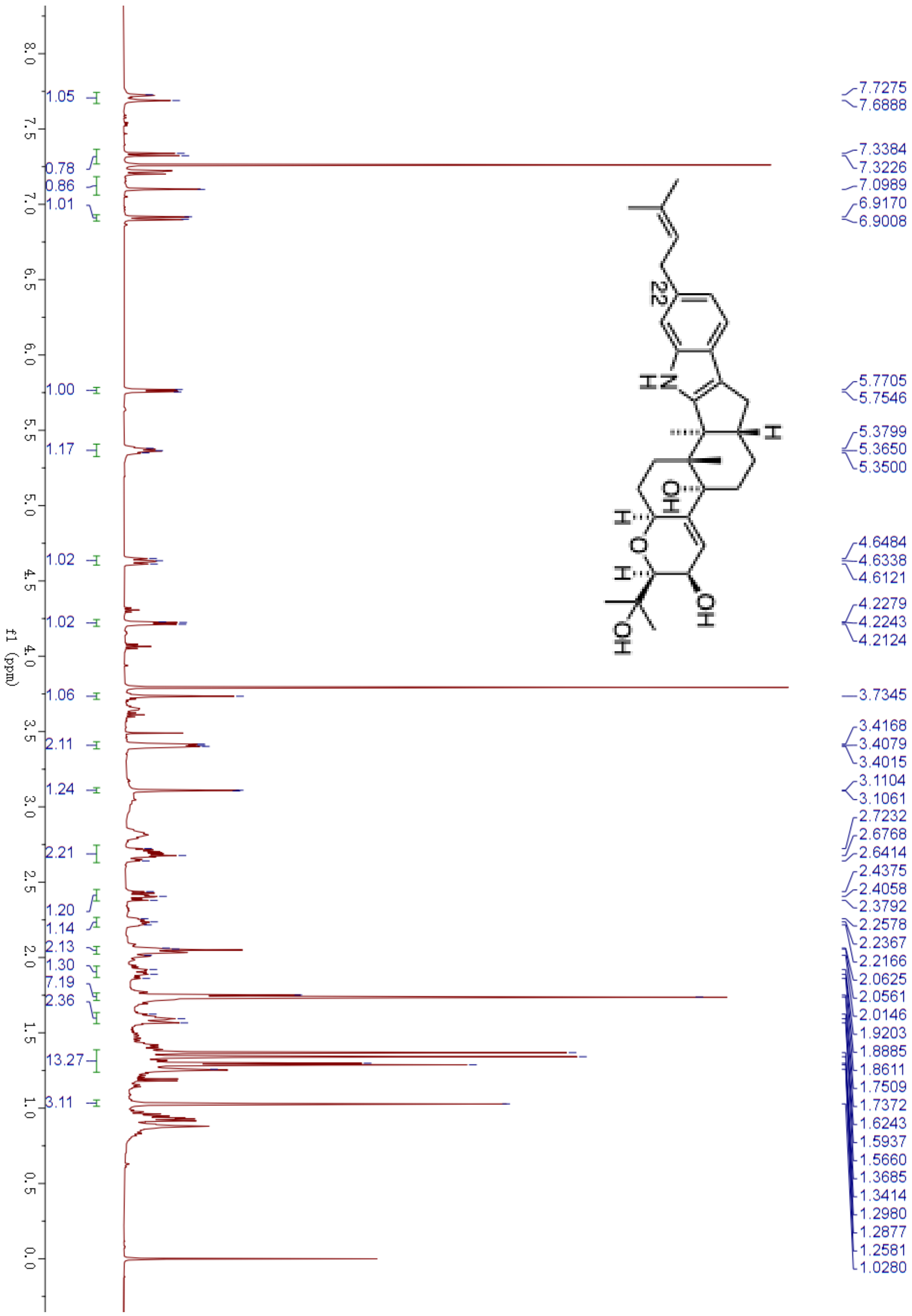

Fig. S23 ^{1}H-NMR of 22-prenylpaxitriol.

References:

Miles CO, Wilkins AL, Gallagher RT, Hawkes AD, Munday SC, Towers NR (1992) Synthesis and tremorgenicity of paxitriols and lolitriol: possible biosynthetic precursors of lolitrem B. J Agric Food Chem 40(2):234-238 https://doi.org/10.1021/jf00014a013

Xu L-L, Hai P, Zhang S-B, Xiao J-F, Gao Y, Ma B-J, Fu H-Y, Chen Y-M, Yang X-L (2019a) Prenylated indole diterpene alkaloids from a mine-soil-derived *Tolypocladium* sp. J Nat Prod 82(2):221-231 https://doi.org/10.1021/acs.jnatprod.8b00589

Xu LL, Pang X-J, Shi Q, Xian P-J, Tao Y-D, Yang X-L (2019b) Two new prenylated indole diterpenoids from *Tolypocladium* sp. and their antimicrobial activities. Chem Biodivers 16(6):e1900116 https://doi.org/10.1002/cbdv.201900116
